# Towards high-quality and timely interim analyses in adaptive trials: a scoping review of best practice and evidence gaps

**DOI:** 10.1186/s13063-026-09643-1

**Published:** 2026-03-21

**Authors:** Katie H. Thomson, Opeyemi Agbeleye, Chizoba Oparah, Alex Inskip, Matthew Breckons, Michelle Bardgett, Alex Bevin-Nicholls, Helen Hancock, Helen Mossop, Julia Phillipson, Dawn Teare, Zoe Walmsley, Nina Wilson, Dawn Craig, James M. S. Wason

**Affiliations:** 1 Population Health Sciences Institute, Faculty of Medical Sciences, Newcastle University, Newcastle Upon Tyne, NE2 4BN UK; 2 NIHR Innovation Observatory, Population Health Sciences Institute, Newcastle University, The Catalyst, Science Square, Newcastle Helix, Newcastle Upon Tyne, NE4 5TG UK; 3 Newcastle Clinical Trials Unit, Population Health Sciences Institute, Newcastle University, 1-4 Claremont Terrace, Newcastle Upon Tyne, NE2 4AE UK

## Abstract

**Background:**

Adaptive designs (ADs) are increasingly used, whereby outcome data accumulated during the trial may inform a trial’s course in accordance with pre-specified rules at interim analyses. The benefits however will only be realised if the approaches to conducting interim analyses are optimised. This study aims to identify existing literature highlighting best practice (and existing evidence gaps) with regard to conducting high-quality, timely interim analyses.

**Methods:**

Medline and Embase databases were searched for studies published between 2005 and July 2025 to identify best practice models and lessons learned for interim analyses in the delivery of adaptive trials. Specifically, we considered papers that discussed adaptive trial methodological approaches (Phases II–IV) in any clinical population and condition. A narrative synthesis focused on the following outcomes was conducted: design considerations, project/trial management, data management, statistical processes, trial committee processes, the implementation of interim decisions and patient and public involvement and engagement (PPIE).

**Results:**

We screened 6720 articles from databases and citation chaining and assessed 329 articles at full text. One hundred and one articles representing 92 unique studies were deemed eligible for inclusion. Key issues included the following: (a) Detailed planning of interim analyses set out in the protocol, and all patient information sheets for possible changes prepared in advance; (b) Effective communication and collaborative decision-making processes across stakeholders, effective staff training, and prompt site query resolutions; (c) Use of electronic data capture with automated data flow processes with integrated query processes to improve data quality; and (d) Use of secure databases with data transfer procedures to maintain data integrity.

**Conclusion:**

The currently available data suggest that although there is a considerable volume of evidence with regards to the conduct of adaptive trials and good trial management, the existing literature base offers limited specific guidance for interim analyses. There are significant gaps in the literature regarding best practice guidance related to PPIE and statistical considerations in adaptive trials. While there are examples of innovative methods speeding up the collation and analysis of data used for interim analyses, we advocate more robust research exploring the operational opportunities and challenges with regards to undertaking adaptive trials.

**Supplementary Information:**

The online version contains supplementary material available at 10.1186/s13063-026-09643-1.

## Introduction

Adaptive designs (ADs) are a type of clinical trial where key aspects of the trial such as treatment allocation, dosing, patient population or sample size can be adjusted based on accumulating data from within the ongoing trial. With increasing costs of trials [1, 2] and the need to answer important research questions rapidly and robustly, ADs have become increasingly prominant in recent years [3]. A recent assessment of the use of adaptive design trials found 236 eligible trials in the Clinical Trial Registry (ClinicalTrials.gov), with trials most frequently used in Phase II and in oncology research [4]. Adaptive clinical trials played a significant role in the research and development of treatments and vaccines for COVID-19, for example the AGILE-ACCORD Platform Trial (EudraCT 2020-001860−27), the RECOVERY Respiratory Support Trial (ISRCTN16912075), the CAPE-Covid and the CAPE-Cod studies (NCT02517489) and the REMAP-CAP Trial (NCT0273570) [5, 6].

The adjustments in ADs are pre-planned and guided by a predefined statistical or algorithmic framework. Adaptive clinical trials offer several potential advantages over traditional fixed-design trials including increased efficiency, flexibility and the ability to more quickly identify effective treatments [7]. However, they also present unique challenges in terms of trial design, implementation and interpretation of results. As such, careful planning and statistical expertise are required to successfully conduct adaptive clinical trials.

Interim analyses involve the periodic evaluations of accumulating data conducted during an adaptive trial. These analyses have pre-specified rules for using the patient outcome data to determine whether (and which) adaptations occur. The benefits that ADs can provide will depend on whether the interim analysis is quick and of high-quality. If the interim analyses take too long, then the efficiency provided by the AD is substantially reduced [8]. This occurs because adaptive trials typically continue recruitment while interim analyses are conducted. Consequently, some participants are enrolled who do not contribute outcome data to the interim analysis and therefore do not influence the adaptive decision-making process. Moreover, these participants do not benefit from any adaptations implemented because of that analysis. If recruitment is paused whilst an interim analysis is underway, then the delay in conducting the interim analyses increases the length the trial takes. Similarly, if the interim analysis is not of high-quality it may risk mistakes being made such as dropping potentially effective treatments. As has been discussed in previous work [8], the negative impact that delay makes depends on the type of AD. ADs that involve potential early stopping of individual arms, or trials, such as multi-arm multi-stage (MAMS) designs are more severely affected by delays than other types such as sample size re-estimation.

Published guidance has been until recently limited to the Food and Drug Administration (FDA) guidelines on adaptive clinical design and conduct [9], and other best practice suggestions on the use and reporting of ADs [3]. More specific guidance has focused on how to adequately resource adaptive trials [10] and on approaches to point estimation [11]. Other perspectives relating to patient representativeness, trial management and data management issues in adaptive platform trials have also been previously described in the academic literature [12–14]. There is however a paucity of evidence on procedures used to ensure the acceptability, quality and speed of interim analyses in ADs.

A scoping review approach will be adopted here to collate and synthesise the evidence related to the analysis and implementation of interim analyses. Scoping reviews are an evidence synthesis approach that aim to map the existing literature on a particular topic, identify key concepts and uncover gaps in knowledge. Unlike systematic reviews, which focus on answering specific research questions using rigorous, predetermined methods, scoping reviews have a broader scope and are more exploratory in nature [15] which is warranted here as the evidence base is evolving. As such this scoping review will aim to identify best practice for conducting high-quality and speedy interim analyses. The following research objectives will be explored:

1. To document how interim analyses are operationalised within clinical trials, particularly focusing on the methodological approaches to data management, statistical procedures and trial management which are employed.
2. To ascertain whether there are any methods, procedures or tools that increase the speed of undertaking interim analyses.
3. To identify the facilitators and barriers to carrying out high quality, speedy and acceptable interim analyses.
4. To highlight any evidence gaps with regards to interim analyses which may inform future research.

## Methods

This study was conducted in accordance with the PRISMA extension for scoping reviews (PRISMA-ScR) [16] (the PRISMA-ScR checklist is detailed in Supplementary Material - Appendix 1) and was pre-registered with the Open Science Framework (https://osf.io/fdsr4/).

### Search strategy

The search strategy was designed in collaboration with an experienced information specialist (AI). Medline and Embase databases were searched from 2005 to 01 June 2023, with an updated search ran on 02 July 2025 (full search strategies are available in Supplementary Material - Appendix 2). Medline and Embase are extensive medically focused bibliographic databases indexing journals in which clinical trial-related material should be found. It would be unexpected for relevant material to be published in journals not covered by one or both. The year limit of 2005 was applied because adaptive design trials have only become prominent in the last 15–20 years [17]. Subject headings and keywords were used as appropriate (and translated across databases accordingly). The search strategy comprised two main elements; firstly, a range of terms covering the concept of platform trials and adaptive design and secondly, aspects of effective implementation of interim analyses. Trials registries themselves (such as clinicaltrials.gov) were not searched directly, as the fields available to search and the data contained therein are not nuanced or detailed enough to be able to identify relevant studies.

In addition to database searches, citation chaining of relevant systematic reviews and included studies was also conducted. We also sought to supplement our published literature searches with statistical analysis plans (SAPs)/trial protocols identified through contacting adaptive trial experts.

### Inclusion/exclusion criteria

#### Participants

As the focus is on the adaptive trial design itself, any clinical population and any condition were considered of interest. We excluded studies not involving human subjects.

#### Concept

Phase II to IV clinical trials on any drug, device or therapeutic or complex intervention were considered if they were adaptive in design (including group-sequential designs) and described approaches for interim analyses. We broadened this definition to include both the analysis and implementation of any resulting decisions about the trial that arise from analysis. Trials were eligible with or without a comparator. Phase I trials were excluded due to their focus on dose-finding and safety, and their distinct statistical and operational contexts, which differ from the structured interim analyses typical of later-phase adaptive trials (Phases II–IV).

#### Context

This scoping review considered studies from all contexts and geographical locations.

#### Types of sources

Only methods-based original research articles in English were eligible for inclusion. These could have related to individual clinical trials (e.g., published trial protocols), or a selection of trials which detail how the interim analyses in AD trials is operationalised. Only research articles, substantive guidance pieces, and protocol papers that described how interim analyses were conducted were eligible for inclusion; brief editorials or purely theoretical simulations were excluded.

Any outcome domains were included if they related to the best practice models and lessons learned for interim analyses in the delivery of adaptive trials. Specifically, we looked at design considerations, project/trial management, data management, statistical processes, trial committee processes, the implementation of interim decisions and patient and public involvement and engagement (PPIE). The scope of each domain is provided in Table 1.

**Table 1:** Scope of domain outcomes used to assess eligibility of included studies

Domain	Scope
Trial committee processes	Setup and remit of trial committees, including composition and their roles and responsibilities
Trial and project management	Planning of key tasks, contingency measures, strategies to engage sites, processes for ensuring critical site queries are resolved prior to data lock and factors that impacted their response to queries
Data management	Efficient processes for ensuring clean data within timelines, data storage including the approaches for updating databases, and extraction
Implementation of interim decisions	Approaches for speedily updating randomisation systems, participant information sheets and trial sites
Design considerations	Timing, indication and number of interim analyses; adaptive features; randomisation; types of design, modes of the design e.g., planned interims
Statistical considerations	Processes for rapid analysis and reporting including automation
PPIE	Consideration of how best to implement PPIE within adaptive trials, perceived barriers /facilitators to its successful implementation

### Screening, data extraction and strategy for data synthesis

Following the removal of duplicates, screening at title and abstract and subsequently full-text was undertaken independently by two reviewers using Rayyan [18]. One reviewer extracted data from the articles and a second reviewer checked the data extraction. Any disagreements in screening or extraction were resolved by discussion, or with the project lead (JW). Extracted information from studies was compiled using Microsoft Excel. Basic data were extracted on the nature of the publication including author name and basic study characteristics (including the year of trial publication or completion date), adaptive design type, phase, type of disease studied in the trial and intervention type (drug, medical device or other).

With regard to the interim analyses specifically, data on methods, procedures and tools highlighted were extracted from the paper and added to an Excel spreadsheet under the following headers: design, project/trial management, data management, statistical considerations, trial committee processes, implementation of interim decisions and PPIE. The data extraction form was pre-piloted on a sample of studies deemed eligible for inclusion in the review, with modifications during the pilot phase allowed to ensure that all relevant information was captured in a time-efficient manner. We present a summary of study characteristics and outcome data, with a narrative commentary collating evidence for best practice approaches/guidance across each.

## Results

**Fig. 1:**
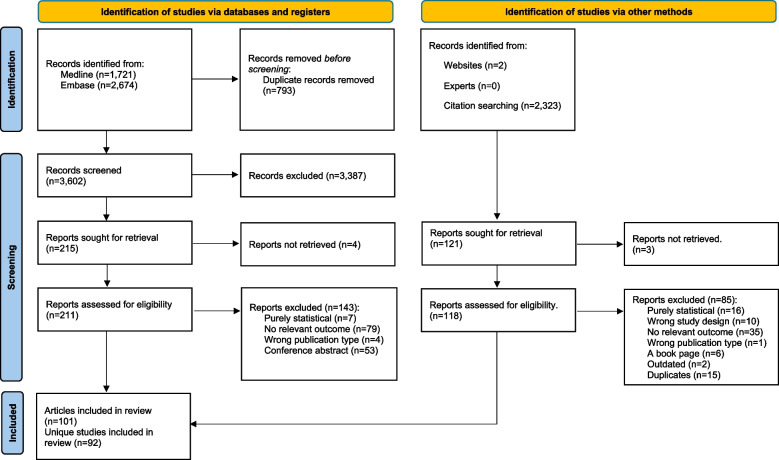
PRISMA flow chart of included studies in scoping review

A total of 4395 references were downloaded from Medline and Embase and following deduplication, 3602 references were remaining (from both the original search and update). Following title/abstract and full-text screening, 68 references were eligible for inclusion (Fig. 1). Two thousand three hundred and twenty-three articles were additionally found during citation chaining; of these, 33 were included. In total, 101 articles met the scoping review inclusion criteria, representing 92 unique studies. The study characteristics of the included studies are fully described in Supplementary Material - Appendix 3, and the counts by domains are shown in Table 2.

**Table 2:** Count of studies reported for each aspect of interim analyses (see Table 1 for description)

Domain outcomes	No. of studies (number of articles)	Article references
Trial committee processes	30 (36)	[3, 13, 14, 19–51]
Project/trial management	17 (22)	[10, 13, 14, 22, 25, 28, 31, 34, 36, 37, 41, 42, 47, 51–59]
Design considerations	64 (72)	[3, 13, 19–24, 26, 30–34, 36, 37, 39, 42–44, 46, 52, 54–57, 60–97]
Data management	68 (76)	[3, 13, 14, 19, 21–37, 39–44, 47–51, 54–58, 60–62, 65–67, 69, 70, 72, 75–79, 81, 82, 86, 89, 91–94, 96, 98–113]
Implementation of interim decisions	19 (22)	[30, 34, 36, 37, 43, 44, 48–50, 54, 66, 79, 81, 85–88, 91, 93–95, 114]
Statistical processes	20 (24)	[24, 30, 37, 42, 46, 48–50, 54–58, 71, 75–78, 81, 87, 88, 99, 100, 115]
PPIE	5 (8)	[10, 13, 14, 20, 32, 51, 59, 82]

*Abbreviations*: *PPIE* patient and public involvement and engagement

**Fig. 2:**
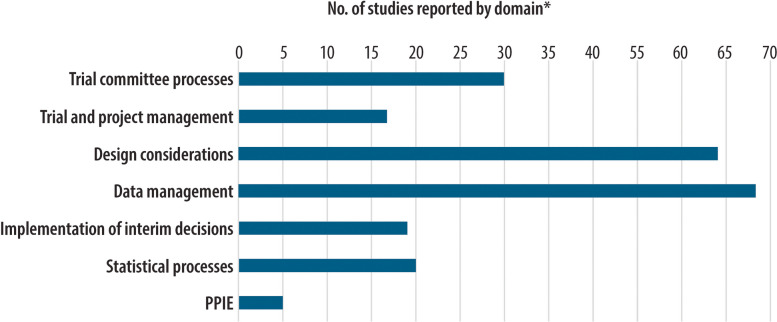
Chart showing the number of studies which incorporated information on each of the seven domains used in the review. *Studies could be coded to multiple domains; hence, the sum of studies is 223 here. Out of the 92 studies included in the review, only 18 reported information regarding one specific aspect of interim analyses, the majority of studies *n* = 74 included reference to multiple domains

Forty-one of the included studies were derived from specific clinical trials, of which 18 were protocols. The clinical trials using an adaptive design encompassed a wide variety of indications including pneumonia, cancer, human papillomavirus, heart disease, childhood immunisation, sepsis, muscle and joint pain and COVID-19. The other 51 included studies were more general overviews of the implementation, design and challenges associated with adaptive trials, including issues related to interim analyses. The papers were published between 2006 and 2025. The 92 included unique studies reported outcomes related to trial committee processes, trial/project management, data management, design considerations, statistical considerations and PPIE (see Fig. 2). Data management issues were the most cited discussion point with regards to interim analyses (*n* = 68), and PPIE was the least (*n* = 5).

### Trial committee processes

Thirty studies (representing 36 articles) explicitly discussed approaches and recommendations for the clear division of trial oversight committee roles and expectations in adaptive trials [3, 13, 19–46]. Most studies reported information related to trial committee structures. The importance of an independent (external) statistician was highlighted in most studies. Their role around the time of interim analyses was raised in Li et al. [35], particularly communicating with the Independent Data Monitoring Committee (IDMC) members the adaptive design implementation procedure before any interim analyses have taken place. Preparing programs to generate IDMC reports and ascertaining the algorithm and decision rules for subsequent sample-size re-estimations was also highlighted as important [46].

Central to this was the role of IDMCs who review interim analyses and safety data and make recommendations on treatment-specific arms [e.g. 21, 22]. Sanchez–Kam et al. [40] noted the importance of experience among committee members in novel adaptive trials, highlighting the need for bespoke (external) training. In addition to the IDMC, Wang et al. [44] and Zheng et al. [46] recommended an Outcome Evaluation Committee. The Outcome Evaluation Committee specifically had the remit to evaluate key outcomes based on clinical expertise, which is particularly important when considering interim analyses.

A number of studies highlighted the importance of charters (which provide a detailed description of clinical trial methods) to clearly set out expectations amongst Committee members [e.g. 3, 19, 25, 29, 38–40, 42, 46]. Specifically referring to interim analyses, Mehta et al. [38] proposed an Interim Analysis Review Committee Charter and Sanchez-Kam et al. [40] describe the importance of an ‘operations plan’. These detail data transfer arrangements, who is undertaking the interim analysis, what the interim analysis report should contain/who has access, and rules and procedures for making adaptations.

Sponsor involvement in interim analyses and related trial committees has been addressed in several studies [e.g. 19, 21, 23, 25, 33, 36, 42, 43, 101]. Although some engagement is unavoidable, maintaining the independence of interim decision-making is critical. Sponsors should not influence data review or analysis outcomes, with autonomy ensured through advance planning or independent IDMCs with broad expertise [33]. Alternative models, such as those described by Spencer et al. [42] included limited sponsor participation via internal review committees, restricted to essential scientific or operational input [42, 101]. Clear communication frameworks and early dialogue among sponsors, regulators and monitoring bodies were also recommended to define boundaries for sponsor involvement in interim analyses [19, 25, 28].

### Project/trial management

Seventeen studies (representing 22 articles) detailed approaches for interim analyses as part of project and trial management [10, 13, 22, 25, 28, 31, 34, 36, 37, 41, 42, 52–57]. These centred on resource use management (particularly on statisticians), operational challenges and site query strategies. The importance of adequate resourcing, planning and setup was identified as particularly important. Most significantly, the availability of an agile, adequately funded Clinical Trials Unit (CTU) with well-trained, in-house experts (trial managers, data managers and statisticians) who were able to be drawn upon in periods of intense activity was highlighted [41, 51] (for example during the addition of a new comparator). More senior-level project management oversight was also recommended to deal with larger staffing numbers and greater scientific and operational complexity compared to a non-adaptive trial [36]. Where possible, the use of existing infrastructure (e.g. a previous related trial) was suggested. For example, the I-SPY 2 adaptive platform breast cancer model was adapted to test promising agents for COVID-19 (I-SPY COVID-19) [52]. Regardless of the trial setup, regular workload prioritisation across the team was identified as important to balance the conflicting tasks of setting up/closing comparisons and ensuring the timely transfer of data [14]. More generally, the importance of early preparation for adaptive changes and the realistic scheduling of tasks was considered paramount [55].

The complexity of the adaptive process and the timing and type of interim analyses warrant staff input to be proportionally larger to reflect additional workload for more complex protocols and SAPs as well as time needed to conduct analyses and communicate findings to the IDMC [25, 59]. The timings of additional statistical support may be difficult to predict and larger CTUs would likely be more adept at dealing with the increased resource required at late notice [41]. A clear understanding of what work could be managed internally and what work should be outsourced is important to ascertain from the outset [42]*.* Staffing shortages were mitigated in some ADs by partnering with a healthcare staffing company to hire off-site data entry specialists [52].

The importance of training and keeping documents up-to-date, particularly as recruiting arms change, was also noted [e.g. 13, 31, 55]. This was emphasised as particularly essential for statisticians [55] to understand the adaptive process, and the extra time needed to prepare and implement interim analyses. Hague et al. [13] (p.13) also reiterated the importance of staff training both at sites and the CTUs and the importance of planning/preparing the update of an increasingly large set of documents both on generic and comparison-specific processes. Operational challenges were also highlighted in included studies. The importance of communication and collaborative decision making across stakeholders (e.g. IDMC, Steering Committee, Statisticians, and sponsor) regarding the adaptive aspects of the trial design was seen as vitally important [34]. In addition, clear and up-to-date documentation to ensure all operational deadlines were met was emphasised in Gaydos et al. [28].

Best practice strategies for working with sites and solving queries were also discussed. From the outset, better coordination of the start-up of new sites was particularly recommended, to minimise data-entry delays [34]. Authors identified the importance of collating data and resolving queries promptly, particularly as analyses may occur close together in ADs. Spencer et al. [42] described their process further: sites had up to 48 h from the study visit to enter data, with review and validation daily. Automatic queries from the data management system and manual queries were then expected to be dealt with promptly (within 48 h). To ensure site queries could be resolved, a clear trial structure was advocated by Love et al. [36] to ensure site teams have a dedicated person/site advisory group for the trial team to contact with questions. In addition, a dedicated central project manager was used to conduct on-site visits for non-compliance [36]. The importance of communication to all relevant site stakeholders was also highlighted. For example, engagement with site pharmacies was encouraged early in order to prepare for future agents before they are added to the trial [52].

### Design considerations

Sixty-four studies (representing 72 articles) detailed various aspects of design considerations [3, 13, 19–24, 26, 30–34, 36, 37, 39, 42–44, 46, 52, 54–57, 60–97]. Studies frequently reported that planning and conducting interim analyses using specified time and randomisation or enrolment schedules can make treatment evaluation more efficient [e.g. 19, 24, 26, 31, 34, 43, 47, 52, 64–66, 73, 84, 89, 94]. Park et al. [97] noted that the frequency of interim evaluations requires careful consideration in order to avoid the risk of false findings which can increase along with the number of analyses, especially without adequate statistical adjustments. Other studies suggest that an important consideration in the timing of interim analyses was the identification of the earliest time point for decisions on study termination due to treatment efficacy or futility, to ensure collection of adequate, valid and reliable patient data thereby enhancing data confidence [e.g. 24, 26, 33, 34, 47, 66, 94].

Studies also reported the mode of designing the interim analyses [e.g. 3, 26, 31–33, 55, 84]. One study reported the appointment of an AD implementer affiliated with the sponsor, whose responsibility involves the oversight of the system interfaces and data transfers between the sponsor and the Independent Statistical Centres (ISCs) to ensure timely reviews of interim analyses [26]. Pallmann et al. [3] noted that it is good practice to prepare patient information sheets and other documents for all possible adaptations at the start of the study. One study highlighted the need to establish and continuously monitor a well-defined cycle time, incorporating data entry, integrity review, and query management processes, to maintain data quality and compliance during interim analyses [55].

Some challenges were reported in planning the conduct of interim analyses: most were based on logistics, time and resource management. Some studies explained that the recruitment criteria, increased documentation, as well as the need for timely rapid data analysis are often resource intensive [e.g. 13, 42, 94]. Falling to plan and acquire adequate resources to address this need could put the quality of results from interim analyses at risk. However, many of the challenges associated with the trial design were addressed by cooperative teamwork between multi-disciplinary partners [46]. Pallmann et al. [3] also highlighted the difficulty in obtaining funding to conduct these adaptive trials as the decision makers are usually not familiar with the methods; however, this can be overcome by ensuring the design was explained in non-technical terms for clarity.

### Data management

Sixty-eight studies (representing 76 articles) reported issues related to data management [3, 13, 14, 19, 21–37, 39–44, 47–51, 54–58, 60–62, 65–67, 69, 70, 72, 75–79, 81, 82, 86, 89, 91–94, 96, 98–113], namely the approaches used to ensure efficient data cleaning, as well as data storage/extraction and challenges facing the conduct of interim analyses. Data cleaning was highlighted as particularly important, with one study stating that data cleaning should be an active, continuous and functional process in order to be easily undertaken [36]. In another study the cleaning process was made more efficient through a reduction of data volume, targeted data use and risk-based streamlined cleaning [36, 66]. In a further study, real-time electronic data capture with adequate planning and resources were reported to make the cleaning process faster and improve data quality [39]. In some studies, automated data flow processes were particularly emphasised, ideally making use of fully integrated systems whereby data from the collection system(s) are extracted in real-time and routed to the adaptive analysis programme [26, 42, 102].

Across the studies, various processes and activities were employed to store extracted data and maintain data integrity. Contingency plans were put in place to ensure preparedness for issues that might occur during data transfer [42]. Methods employed to ensure the integrity of the data included the use of a secure file transfer service, regular data monitoring performed remotely using an electronic application support, or performed manually by trained personnel, and databases locked when necessary [24, 31, 42, 55, 72, 96]. Studies reported that data collection and extraction were efficiently carried out using various automated platforms consisting of electronic systems that allowed data to be collected directly (e.g. Peek Vision data collection and analysis software which utilises prewritten R scripts) [24, 26, 31, 44, 72, 82, 86, 89]. Integrated in these automated systems were query processes to support monitoring and resolution of errors during data collection [26, 31]. In another study, where automation was not used for queries, ample time was given to resolving any missing data forms [13].

Most studies reported challenges related not only to interim analyses but to overall data management, including staffing needs due to high data volumes, extended database testing following design changes, variable staff experience with electronic data capture systems, and difficulties integrating data management systems [e.g. 13, 19, 42, 51, 72]. To mitigate some of these challenges, studies employed various methods which included the following: converting the database into modular ones thereby gaining time by testing only the affected modular databases whenever there were modifications to the trial, implementation of user-friendly systems in studies involved with site staff with varied experiences in data capturing, and the use of descriptive analytic dashboards that prompt users to complete data input and alerted users to missed responses to reduce the likelihood of missing data [13, 24, 51, 72]. In relation to interim analyses specifically, the prioritisation of data cleaning by the time of each interim analysis was also highlighted [41]. The use of a standardised data resolution workflow module was suggested by Hager et al. [31] to specifically query omitted data or data found to be inaccurate/inconsistent.

### Implementation of interim decisions

Nineteen studies (representing 22 articles) reported data related to implementation of interim decisions [30, 34, 36, 37, 43, 44, 48–50, 54, 66, 79, 81, 85–88, 91, 93–95, 114]. Most studies reported that decisions to stop or continue trials following interim analyses were made by the IDMC, although one study noted sponsor involvement in this process [42]. Recognising the complexity of interim decision-making, one study recommended detailed advance planning and the provision of explicit guidance to decision-makers to promote efficient implementation [68].

### Statistical considerations

Twenty studies (representing 24 articles) highlighted the importance of automating the statistical analysis process in response to interim data [24, 30, 37, 42, 46, 48–50, 54–58, 71, 75–78, 81, 87, 88, 99, 100, 115]. Many studies mentioned that statistical analysis and/or report writing could be automated as part of the trial operating procedure, typically alongside the routine data collection from trial sites and strict data cleaning/quality checks. Although the extent of automation was unclear, Spencer et al. [42] highlighted the importance of timely analysis in their trial for a diabetic therapeutic, highlighting reports were sent from the Safety Assessment Committee to the IDMC every two weeks. Authors also highlighted that automation was particularly beneficial in trials which use response-adaptive randomisation where frequent interim updates are needed [71]. Studies mentioned the importance of prepopulating interim analyses reports with mock-up tables, listings and graphs so analysis could be prompt following the receipt of interim data [e.g. 46, 55, 76, 100]. Dey and Pyle [76] recommended a standard operating procedure for interim analyses, comprising the roles of all stakeholders, timelines, data collection, programme development, validation, testing and execution (p. 2).

#### PPIE

Five studies (representing eight articles) discussed the use of PPIE in adaptive trials [14, 20, 32, 59, 82]. While two stressed the importance of public members in the Trial Management Group from the initial trial planning meetings onwards [14, 20], these centred primarily on intervention development [20, 82]. One study reported the additional oversight meetings linked to discussing interim analyses had budget implications for PPIE contributors [59]. Another study reported that seven people in their target recruitment age group reviewed the patient-facing materials, including outcomes and delivery plans [32]. No specific mention of the role of PPIE in the planning or the execution of interim analyses was identified.

## Discussion

**Fig. 3:**
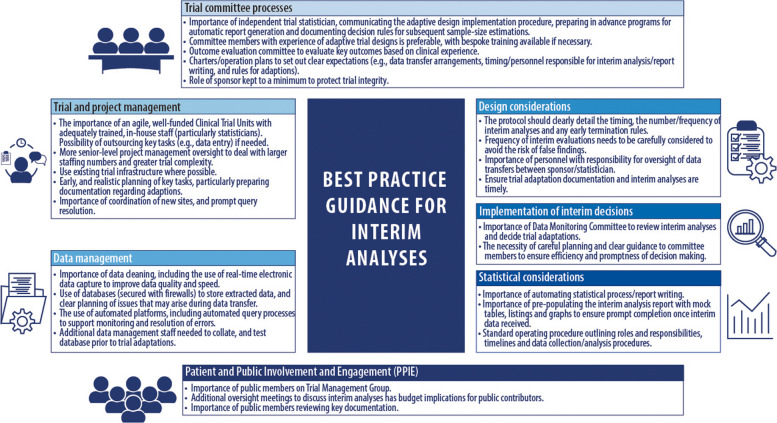
Infographic summarising key best practice recommendations across the domains (domains created by authors for this review)

This scoping review has synthesised content from 92 studies (representing 101 articles) and is to our knowledge the first scoping review to discuss best practice guidance for high-quality and timely interim analyses in adaptive trials. We present a comprehensive overview of practical approaches different groups have suggested/used which may help facilitate interim analyses. Importantly, we have not evaluated the approaches; rather, we have presented these and organised them across seven key outcome domains. Previous reviews have focused on technical, statistical and regulatory aspects of the implementation of clinical trials [12, 116], and were not specifically focused on guidance/best practice for interim analyses. This scoping review sought to describe approaches for interim analyses in adaptive trials across several different areas. A visual summary of the review findings can be found in Fig. 3, highlighting key best practice recommendations across the review domains.

Findings from the reviewed literature indicate that many studies, both trials and commentary articles, address specific aspects of interim analyses in adaptive clinical trials. However, the depth of reporting regarding interim analyses varied substantially across the 92 studies, ranging from detailed methodological discussions to general statements offering limited insight. In terms of the most prominent domains identified, there was a considerable body of evidence pertaining particularly to trial and project management, and data management. Many of the included studies advocated for agile trial teams, with available resources (either in-house or off-site) brought in to help capture and analyse data needed for interim analyses. The importance of maintaining good relations with trial sites, particularly when new sites are added, was also highlighted. Several best practice approaches were discussed in relation to data management. Successful approaches adopted included real-time electronic data capture, data transfer services and bespoke databases with strong firewalls built in to preserve the integrity of trial results.

There are significant gaps in the literature regarding best practice on some statistical considerations, namely the practicalities of accessing data and performing planned analyses as well as the use of PPIE in adaptive trials. Our review highlighted that detailed interim analysis workflows are rarely published, as they are often governed by internal standard operating procedures (SOPs) that remain inaccessible to the broader research community. In addition, few research groups may consider such workflows to be suitable for publication or view them as worthwhile to develop for inclusion as supplementary materials in other papers. We encourage investigators to document their interim analysis conduct in publications or share protocols/SOPs, to build a knowledge base for others to learn from, even when trials encounter difficulties. Operational approaches should routinely be discussed and ideally be included in future reporting frameworks to promote transparency and encourage wider uptake of best practices in adaptive trials.

Only five papers were identified highlighting the importance of PPIE methods specifically in the context of adaptive trials. In clinical trials more generally, there has been a recent recognition of best practice approaches to integrate patients in the design and conduct of clinical trials, including systematic reviews synthesising the available evidence [117]. While studies have demonstrated PPIE was likely to improve enrolment and retention in clinical trials [118], there remains widespread recognition that PPIE should be embedded early and PPIE contributors and researchers should have managerial roles rather than being limited to oversight-only positions [119]. In trial publications, PPIE is often not reported adequately [120], and thus specific toolkits have been developed for meaningful and flexible involvement in clinical trials [121]. In adaptive trials specifically, there has been no clear overview of how best patient involvement should be integrated in the trial process, and particularly how to manage some of the concerns for patients regarding trial arms stopping for lack of benefit and adding research arms mid-trial. PPIE work conducted alongside this review highlighted the importance of integrating PPIE early in the planning process [122]. Public partners can support research teams by ensuring trial procedures are acceptable and by helping communicate sensitively with participants, particularly the dropping or adding of treatment arms.

Our scoping review had many strengths. The review has been undertaken in line with guidance from the PRISMA extension for scoping reviews [16]. Two databases were comprehensively searched, alongside forward and back citation chaining of most of the studies. Screening was undertaken in duplicate, and extraction was undertaken by one reviewer and checked in full by a second. Although the approaches adopted were robust, only papers that specifically mentioned operational challenges in the title/abstract were taken through to full-text review. It was possible that some papers which were primarily focused on trial results would have been excluded if details of operational challenges were only listed in the main body of the paper (and not the title/abstract). The review also didn’t consider the role of trial sponsorship. The use of ADs in clinical trials is influenced not only by their scientific advantages but also by practical considerations. Industry-funded trials often have greater resources to implement complex ADs, whereas investigator-initiated trials may face limitations affecting interim analyses and design choice. Future research may consider examining this relationship further.

As a deviation to the protocol, citation chaining was only undertaken over two rounds of studies deemed eligible. Following other scoping reviews [123], we used the principle of saturation such that citation chaining was complete when no new suggestions or approaches were identified. In addition, for logistical reasons, it was not possible to review all adaptive trial protocols/SAPs to explore strategies for interim analyses. Although targeted emails were sent to relevant experts and groups, no protocols/SAPs were retrieved. We also did not assess the quality of reports, as the data were collated from both specific trials and other publications pertaining to a range of clinical trials.

## Conclusion

The use of adaptive trials has substantial potential to improve the efficiency and speed of clinical research. However, this potential is highly dependent on the speed and quality of interim analyses. The currently available data suggest that while there is a considerable volume of evidence with regards to the conduct of adaptive trials and good trial management generally, the literature to date offers limited specific guidance for interim analyses. Although there are examples of innovative methods speeding up the collation and analysis of data used for interim analyses, we would encourage more academic publications exploring the operational opportunities and challenges with regards to undertaking adaptive trials. The reporting of approaches to the operationalisation of interim analyses in the methods section of clinical trial reports, or specific academic publications, could aid replication and make approaches available for others to adapt and refine. In addition, more research needs to explore how the public and patient voice can be best integrated in adaptive trials, particularly helping inform trial participants of changes in trial design following interim analyses. Lastly, the findings of this scoping review also highlight the need for the development of guidance, which has since been published by the wider research team [124] and details recommendations by stage of trial (e.g., planning stages, trial setup, and then pre-, during and post-interim analysis). 

## Supplementary Information

Supplementary Material 1.

## Data Availability

The datasets used and/or analysed during the current study are available from the corresponding author on reasonable request.

## Abbreviations

AD: Adaptive designs
CTU: Clinical Trials Unit
FDA: Food and Drug Administration
IDMC: Independent Data Monitoring Committee
ISCs: Independent Statistical Centres
NA: Not specified
NR: Not reported
PPIE: Patient and Public Involvement and Engagement
PRISMA-ScR: Preferred Reporting Items for Systematic reviews and Meta-Analyses extension for Scoping Reviews
REDCap: Research Electronic Data Capture
SAPs: Statistical analysis plans
