# Supplementary material for: Towards high-quality and timely interim analyses in adaptive trials: a scoping review of best practice and evidence gaps
Source: Trials. 2026 Mar 21;27:335. doi: 10.1186/s13063-026-09643-1 (PMC13130667; doi:10.1186/s13063-026-09643-1)
Supplement: Supplementary file 1 — Supplementary Material 1. [file 13063_2026_9643_MOESM1_ESM.docx]

**Supplementary Appendix 1**

**Preferred Reporting Items for Systematic reviews and Meta-Analyses extension for Scoping Reviews (PRISMA-ScR) Checklist**

| **SECTION** | **ITEM** | **PRISMA-ScR CHECKLIST ITEM** | **REPORTED ON PAGE # (sections)** |
| --- | --- | --- | --- |
| **TITLE** | | | |
| Title | 1 | Identify the report as a scoping review. | Manuscript title |
| **ABSTRACT** | | | |
| Structured summary | 2 | Provide a structured summary that includes (as applicable): background, objectives, eligibility criteria, sources of evidence, charting methods, results, and conclusions that relate to the review questions and objectives. | Abstract |
| **INTRODUCTION** | | | |
| Rationale | 3 | Describe the rationale for the review in the context of what is already known. Explain why the review questions/objectives lend themselves to a scoping review approach. | Introduction |
| Objectives | 4 | Provide an explicit statement of the questions and objectives being addressed with reference to their key elements (e.g., population or participants, concepts, and context) or other relevant key elements used to conceptualize the review questions and/or objectives. | Introduction |
| **METHODS** | | | |
| Protocol and registration | 5 | Indicate whether a review protocol exists; state if and where it can be accessed (e.g., a Web address); and if available, provide registration information, including the registration number. | Methods |
| Eligibility criteria | 6 | Specify characteristics of the sources of evidence used as eligibility criteria (e.g., years considered, language, and publication status), and provide a rationale. | Methods |
| Information sources* | 7 | Describe all information sources in the search (e.g., databases with dates of coverage and contact with authors to identify additional sources), as well as the date the most recent search was executed. | Methods |
| Search | 8 | Present the full electronic search strategy for at least 1 database, including any limits used, such that it could be repeated. | Supplementary Appendix 2 |
| Selection of sources of evidence† | 9 | State the process for selecting sources of evidence (i.e., screening and eligibility) included in the scoping review. | Methods |
| Data charting process‡ | 10 | Describe the methods of charting data from the included sources of evidence (e.g., calibrated forms or forms that have been tested by the team before their use, and whether data charting was done independently or in duplicate) and any processes for obtaining and confirming data from investigators. | Methods |
| Data items | 11 | List and define all variables for which data were sought and any assumptions and simplifications made. | Table 1 |
| Critical appraisal of individual sources of evidence§ | 12 | If done, provide a rationale for conducting a critical appraisal of included sources of evidence; describe the methods used and how this information was used in any data synthesis (if appropriate). | Not completed |
| Synthesis of results | 13 | Describe the methods of handling and summarizing the data that were charted. | Methods |
| **RESULTS** | | | |
| Selection of sources of evidence | 14 | Give numbers of sources of evidence screened, assessed for eligibility, and included in the review, with reasons for exclusions at each stage, ideally using a flow diagram. | Figure 1 |
| Characteristics of sources of evidence | 15 | For each source of evidence, present characteristics for which data were charted and provide the citations. | Supplementary Appendix 3 |
| Critical appraisal within sources of evidence | 16 | If done, present data on critical appraisal of included sources of evidence (see item 12). | Not completed |
| Results of individual sources of evidence | 17 | For each included source of evidence, present the relevant data that were charted that relate to the review questions and objectives. | Table 2 and Supplementary Appendix 3 |
| Synthesis of results | 18 | Summarize and/or present the charting results as they relate to the review questions and objectives. | Results |
| **DISCUSSION** | | | |
| Summary of evidence | 19 | Summarize the main results (including an overview of concepts, themes, and types of evidence available), link to the review questions and objectives, and consider the relevance to key groups. | Discussion |
| Limitations | 20 | Discuss the limitations of the scoping review process. | Discussion |
| Conclusions | 21 | Provide a general interpretation of the results with respect to the review questions and objectives, as well as potential implications and/or next steps. | Discussion |
| **FUNDING** | | | |
| Funding | 22 | Describe sources of funding for the included sources of evidence, as well as sources of funding for the scoping review. Describe the role of the funders of the scoping review. | Funding statement |

JBI = Joanna Briggs Institute; PRISMA-ScR = Preferred Reporting Items for Systematic reviews and Meta-Analyses extension for Scoping Reviews.

* Where *sources of evidence* (see second footnote) are compiled from, such as bibliographic databases, social media platforms, and Web sites.

† A more inclusive/heterogeneous term used to account for the different types of evidence or data sources (e.g., quantitative and/or qualitative research, expert opinion, and policy documents) that may be eligible in a scoping review as opposed to only studies. This is not to be confused with *information sources* (see first footnote).

‡ The frameworks by Arksey and O’Malley (6) and Levac and colleagues (7) and the JBI guidance (4, 5) refer to the process of data extraction in a scoping review as data charting*.*

§ The process of systematically examining research evidence to assess its validity, results, and relevance before using it to inform a decision. This term is used for items 12 and 19 instead of "risk of bias" (which is more applicable to systematic reviews of interventions) to include and acknowledge the various sources of evidence that may be used in a scoping review (e.g., quantitative and/or qualitative research, expert opinion, and policy document).

*From:* Tricco AC, Lillie E, Zarin W, O'Brien KK, Colquhoun H, Levac D, et al. PRISMA Extension for Scoping Reviews (PRISMAScR): Checklist and Explanation. Ann Intern Med. 2018;169:467–473.

**Supplementary Appendix 2**

Literature searches – run 1^st^ June 2023*

Ovid MEDLINE(R) and Epub Ahead of Print, In-Process, In-Data-Review & Other Non-Indexed Citations, Daily and Versions <1946 to May 31, 2023>

| 1 | adaptive clinical trial/ or Adaptive Clinical Trials as Topic/ or adaptive design.kf. | 569 |
| --- | --- | --- |
| 2 | ((adaptive adj4 (trial or trials)) or ((adaptive adj2 (study or studies or protocol$)) and ((clinical$ adj2 stud$) or trial$ or biomarker$ or subgroup$))).mp. | 1841 |
| 3 | (trial$ platform$ or platform trial$ or platform clinical trial$ or platform protocol$ or ((platform design or platform study or platform studies) and (trial$ or clinical$))).mp. | 715 |
| 4 | (((adaptive adj2 design$) and ((clinical$ and (study or studies or protocol$)) or biomarker$ or treatment$ or subgroup$)) or ((adaptive adj6 design$) and (trial or trials))).mp. or (flexible design.kw. and (trial or trials).mp.) | 2261 |
| 5 | (adaptive platform$ and (trial or trials)).mp. | 98 |
| 6 | (complex innovative adj3 (trial$ or design$ or protocol$)).mp. | 19 |
| 7 | master protocol$.mp. | 260 |
| 8 | response adaptive randomi$.mp. | 145 |
| 9 | ((multi arm or multiarm) and (multi stage or multistage)).mp. | 133 |
| 10 | (trial$ adj5 (multi stage or multistage)).mp. | 177 |
| 11 | ((bayesian adj3 design$) and ((clinical$ and (study or studies or protocol$)) or biomarker$ or treatment$ or trial$)).mp. | 722 |
| 12 | ((biomarker$ guided or biomarker$ driven or (biomarker$ adj4 subgroups)) and (biomarker adj5 (trial$ or design$))).mp. | 353 |
| 13 | (drop the loser$ or pick the winner$).mp. | 90 |
| 14 | or/1-13 | 4798 |
| 15 | (((interim or intermediate) adj5 analy$).mp. and (Adaptive Clinical Trials as Topic/ or methods/ or research design/ or exp Clinical Trials as Topic/mt)) or interim analysis.kf. | 897 |
| 16 | (data adj3 (manag$ or workflow or sharing)).mp. | 30322 |
| 17 | financial management/ or exp budgets/ or Resource Allocation/ or "personnel staffing and scheduling"/ or workload/ or financial support/ or healthcare financing/ or research support as topic/ or Capital Financing/ or funding.ti,kf. | 112695 |
| 18 | *adaptive clinical trial/ or *Adaptive Clinical Trials as Topic/ | 65 |
| 19 | *research design/ | 43366 |
| 20 | *methods/ | 983 |
| 21 | (((trial$ or design or interim or analy$ or increas$ or improv$) adj3 efficien$) or efficiencies).mp. or efficien$.ti,kw. | 291900 |
| 22 | efficien$.ab. /freq=3 | 90126 |
| 23 | ((complexit$ or guidance or guidelines or recommendations or considerations or issues or obstacles or barriers) and (trial$ or design$ or analy$ or interim)).ti. | 16887 |
| 24 | (lesson or lessons or pitfall or pitfalls).mp. | 122933 |
| 25 | (trial$ adj4 (manag$ or run or running)).mp. | 8704 |
| 26 | ((good or best) adj3 practice$).ti,kf. or ((good practice$ or best practice$) adj5 (adaptive or interim or design$)).ab. | 11891 |
| 27 | exp *Clinical Trials as Topic/mt | 13750 |
| 28 | ((stop$ or halt$ or paus$ or drop$) adj5 (arm or arms)).mp. | 703 |
| 29 | (decision$ adj2 algorithm$).mp. | 3093 |
| 30 | (costing or costed or resourcing or ((allocat$ or manag$ or requir$) adj4 (cost$ or resource$ or staff$)) or (trial$ adj2 cost$)).mp. or (costs or costing).ti. | 140610 |
| 31 | ((infrastructur$ or operational or practical or financial or procedural or methodolog$ or statistical or design$ or interim or analy$) adj4 (complexit$ or guidance or guidelines or recommendations or considerations or issues or challenges or obstacles or barriers or difficulties or advantages or disadvantages or limitations or utility or flexibility)).mp. | 191679 |
| 32 | (trial$ and (design$ or rationale$ or implement$ or methodol$)).ti. and (Endpoint Determination/ or "early termination of clinical trials"/ or patient selection/ or Medical Futility/ or efficien$.mp.) | 2015 |
| 33 | (compar$ adj3 method$).mp. and (method$.ti. or methods/ or research design/ or Adaptive Clinical Trials as Topic/ or (interim or intermediate or efficien$).mp.) | 42962 |
| 34 | seamless.mp. and (Adaptive Clinical Trials as Topic/ or methods/ or research design/ or efficien$.mp.) | 1027 |
| 35 | (staffing or operational or conduct or conducting).ti. | 24946 |
| 36 | operational.ab. /freq=3 | 2031 |
| 37 | or/15-36 | 1026148 |
| 38 | 14 and 37 | 2045 |
| 39 | limit 38 to yr="2005 -Current" | 1969 |
| 40 | 39 and statist*.jw. | 644 |
| 41 | 39 not statist*.jw. | 1325 |

<https://ovidsp.ovid.com/ovidweb.cgi?T=JS&NEWS=N&PAGE=main&SHAREDSEARCHID=3ToIl8694A3GXO0tSvI2to0UMrSwSLROGDJHZILMaqXdbHr1aX6Iyqtdsd2ZUtGDp>

Embase <1974 to 2023 May 31> *

| 1 | adaptive clinical trial/ or "adaptive clinical trial (topic)"/ or adaptive design.dj,kf. | 1236 |
| --- | --- | --- |
| 2 | ((adaptive adj4 (trial or trials)) or ((adaptive adj2 (study or studies or protocol$)) and ((clinical$ adj2 stud$) or trial$ or biomarker$ or subgroup$))).mp. | 3217 |
| 3 | (trial$ platform$ or platform trial$ or platform clinical trial$ or platform protocol$ or ((platform design or platform study or platform studies) and (trial$ or clinical$))).mp. | 1480 |
| 4 | (((adaptive adj2 design$) and ((clinical$ and (study or studies or protocol$)) or biomarker$ or treatment$ or subgroup$)) or ((adaptive adj6 design$) and (trial or trials))).mp. or (flexible design.kw. and (trial or trials).mp.) | 3669 |
| 5 | (adaptive platform$ and (trial or trials)).mp. | 164 |
| 6 | (complex innovative adj3 (trial$ or design$ or protocol$)).mp. | 37 |
| 7 | master protocol$.mp. | 487 |
| 8 | response adaptive randomi$.mp. | 228 |
| 9 | ((multi arm or multiarm) and (multi stage or multistage)).mp. | 236 |
| 10 | (trial$ adj5 (multi stage or multistage)).mp. | 260 |
| 11 | ((bayesian adj3 design$) and ((clinical$ and (study or studies or protocol$)) or biomarker$ or treatment$ or trial$)).mp. | 1260 |
| 12 | ((biomarker$ guided or biomarker$ driven or (biomarker$ adj4 subgroups)) and (biomarker adj5 (trial$ or design$))).mp. | 655 |
| 13 | (drop the loser$ or pick the winner$).mp. | 192 |
| 14 | or/1-13 | 8621 |
| 15 | (((interim or intermediate) adj5 analy$).mp. and ("adaptive clinical trial (topic)"/ or study design/ or methodology/)) or interim analysis.dj. | 1227 |
| 16 | (data adj3 (manag$ or workflow or sharing)).mp. | 43072 |
| 17 | financial management/ or budget/ or resource management/ or resource allocation/ or workload/ or *funding/ or funding.ti,kf. | 243747 |
| 18 | *adaptive clinical trial/ or *"adaptive clinical trial (topic)"/ | 207 |
| 19 | *study design/ | 3719 |
| 20 | *methodology/ | 23697 |
| 21 | (((trial$ or design or interim or analy$ or increas$ or improv$) adj3 efficien$) or efficiencies).mp. or efficien$.ti,kw. | 337979 |
| 22 | efficien$.ab. /freq=3 | 102588 |
| 23 | ((complexit$ or guidance or guidelines or recommendations or considerations or issues or obstacles or barriers) and (trial$ or design$ or analy$ or interim)).ti. | 20839 |
| 24 | (lesson or lessons or pitfall or pitfalls).mp. | 152306 |
| 25 | (trial$ adj4 (manag$ or run or running)).mp. | 13935 |
| 26 | ((good or best) adj3 practice$).ti,kf. or ((good practice$ or best practice$) adj5 (adaptive or interim or design$)).ab. | 17059 |
| 27 | *statistical design/ or *statistical analysis/ | 20203 |
| 28 | ((stop$ or halt$ or paus$ or drop$) adj5 (arm or arms)).mp. | 1498 |
| 29 | (decision$ adj2 algorithm$).mp. | 4435 |
| 30 | (costing or costed or resourcing or ((allocat$ or manag$ or requir$) adj4 (cost$ or resource$ or staff$)) or (trial$ adj2 cost$)).mp. or (costs or costing).ti. | 202256 |
| 31 | ((infrastructur$ or operational or practical or financial or procedural or methodolog$ or statistical or design$ or interim or analy$) adj4 (complexit$ or guidance or guidelines or recommendations or considerations or issues or challenges or obstacles or barriers or difficulties or advantages or disadvantages or limitations or utility or flexibility)).mp. | 248346 |
| 32 | (trial$ and (design$ or rationale$ or implement$ or methodol$)).ti. and (bioassay/ or patient selection/ or drug dose reduction/ or drug withdrawal/ or early termination of clinical trial/ or efficien$.mp.) | 2410 |
| 33 | (compar$ adj3 method$).mp. and (method$.ti. or methodology/ or study design/ or "adaptive clinical trial (topic)"/ or (interim or intermediate or efficien$).mp.) | 61498 |
| 34 | seamless.mp. and (methodology/ or study design/ or "adaptive clinical trial (topic)"/ or efficien$.mp.) | 1604 |
| 35 | (staffing or operational or conduct or conducting).ti. | 27010 |
| 36 | operational.ab. /freq=3 | 2800 |
| 37 | or/15-36 | 1352289 |
| 38 | 14 and 37 | 2672 |
| 39 | limit 38 to yr="2005 -Current" | 2604 |
| 40 | 39 and statist$.jx. | 526 |
| 41 | 39 not statist$.jx. | 2078 |

<https://ovidsp.ovid.com/ovidweb.cgi?T=JS&NEWS=N&PAGE=main&SHAREDSEARCHID=1w5Vwio2vY2DeaqXvrXUjedEsNST9BrTHpI9gZlU6dUWVlkbplDBtXaLbmZf6RJDs>

* Search results in Medline and Embase were rerun on 02 July 2025. Results were exported and deduplicated – against each other (Medline/Embase), and also against the original/updated results. Embase now includes clinicaltrials.gov records, which it did not at the time the original searches were run. These results were excluded for the same reason clinicaltrials.gov itself was not searched originally – the difficulty in distinguishing relevant material based on the fields available to search.

**Supplementary Appendix 3 (overleaf)**

| **No.** | **Author**  Lead paper listed in bold (subsequent linked papers are also listed) | **Article type** | **Trial name (if applicable), phase (registration) and trial start date** | **Trial description (where focus is on a trial)** | **Aspect(s) of interim analyses considered** | | | | | | |
| --- | --- | --- | --- | --- | --- | --- | --- | --- | --- | --- | --- |
|  |  |  |  |  | **Trial committee processes** | **Project/trial management** | **Design considerations** | **Data management** | **Implementation of interim decisions** | **Statistical processes** | **PPIE** |
| 1 | **The Adaptive Platform Trials (APTs) Consortium 2019 [1]** | Research article (discusses the use of APTs, and reviews standard features and issues that arise with such trials, offering recommendations to promote best practices in their design, conduct, oversight, and reporting). | NA |  |  |  | ✔ |  |  | ✔ |  |
| 2 | **Aryal et al. 2021 [2]**  Huang 2021[3] | Research article (discusses implementation of trial). | REMAP-CAP, Phase III, NCT02735707, April 2016 | Operationalisation and implementation of Randomised, embedded, multifactorial, adaptive platform trial to evaluate the effect of a range of interventions to improve the outcome of patients admitted to intensive care with community-acquired pneumonia and adapted for COVID-19. |  |  | ✔ | ✔ |  |  |  |
| H3 | **Bhatt and Mehta 2016 [4]** | Research article (focusses on adaptive designs of confirmatory clinical trials and the benefits and limitations of such designs, using studies that highlight the statistical and operational considerations). | NA |  |  |  | ✔ |  |  |  |  |
| 4 | **Bauer et al. 2015 [5]** | Research article (discusses opportunities and pitfalls of confirmatory adaptive designs). | NA |  | ✔ |  | ✔ | ✔ |  |  |  |
| 5 | **Bender et al. 2023 [6]** | Research article discussing adaptive design methods to accelerate adoption of a digital asthma management intervention. | NA |  | ✔ |  | ✔ |  |  |  | ✔ |
| 6 | **Bretz et al. 2009 [7]** | Research article (discusses implementation of adaptive designs in drug discovery). | NA |  | ✔ |  | ✔ | ✔ |  |  |  |
| 7 | **Brown et al. 2023 [8]** | Research article (discusses comparative effectiveness of adaptive design in drug discovery). | NA |  |  |  | ✔ |  |  |  |  |
| 8 | **Bwakura et al. 2024 [9]** | Study protocol | Co-SAM, Phase III, NCT05994742, July 2024 | An adaptive multi-arm trial to improve clinical outcomes among children recovering from complicated severe acute malnutrition. |  |  |  | ✔ |  |  |  |
| 9 | **Chen et al. 2025 [10]** | Research article (discusses a freely accessible tool that provides effective and convenient visualisation tool for basket trials with interim analysis: RaBIt). | NA |  |  |  |  | ✔ |  | ✔ |  |
| 10 | **Chen et al. 2015 [11]** | Research article (discusses design rationale, and implementation of trial). | V503-001, Phase IIB/II, September 2007 | Randomised, double-blinded, dose-ranging, safety, immunogenicity and efficacy study of a multivalent Human Papillomavirus (HPC) L1 virus-like particle vaccine. | ✔ | ✔ | ✔ | ✔ |  |  |  |
| 11 | **Chow et al. 2012 [12]** | Research article describing the independence of data monitoring committees in adaptive trials. | NA |  |  |  |  | ✔ |  | ✔ |  |
| 12 | **Cook et al. 2006. [13]** | Research article (discusses flexibility of clinical trial design). | WIZARD, Phase NS, October 1997 | Randomised, double-blind, group-sequential, placebo-controlled trial assessing the efficacy of azithromycin in preventing progression of clinical coronary artery disease who had a myocardial infarction. | ✔ |  | ✔ | ✔ |  |  |  |
| 13 | **Currie et al. 2023 [14]** | Study protocol | AuTOMATIC, Phase NS, ACTRN12618000789268, registered May 2018 | Adaptive randomised trial aiming to evaluate and optimise the use of short message service (SMS) reminders to improve childhood immunisation coverage. | ✔ |  | ✔ | ✔ |  | ✔ |  |
| 14 | **Dawson et al. 2024 [15]** | Research article (discusses description of adaptive trials and operationalisation of interim analysis). | NOTACS, NCT05308719 (ISRCTN14092678), September 2020 | A multi-centred randomised controlled trial to assess the effect of high-flow nasal therapy on patient-centred outcomes in patients at high risk of postoperative pulmonary complications after cardiac surgery. |  |  | ✔ | ✔ |  | ✔ |  |
| 15 | **Demets and Wittes 2021 [16]** | Research article (discusses approaches to improving the quality of reports to data monitoring committees). | NA |  | ✔ | ✔ |  | ✔ |  |  |  |
| 16 | **Dey and Pyle 2008 [17]** | Overview article (discusses the management of operational and programming challenges of data analysis in interim analyses). | NA |  |  |  | ✔ | ✔ |  | ✔ |  |
| 17 | **Fabbri et al. 2024 [18]** | Overview article (design and implementation approaches/challenges with respect to adaptive trials). | NA |  |  |  | ✔ | ✔ |  | ✔ |  |
| 18 | **Fardipour et al. 2009 [19]** | Overview article (design and implementation approaches/ challenges with respect to adaptive trials). | NA |  | ✔ |  | ✔ | ✔ |  |  |  |
| 19 | **Files et al. 2022. [20]** | Study protocol | I-SPY COVID, Phase II, NCT04488081, March 2024 | Multi-centre, open-label platform trial to evaluate therapeutics that may have a large effect on improving outcomes from severe COVID-19. |  | ✔ | ✔ |  |  |  |  |
| 20 | **Fleming et al. 2024 [21]** | Research article (discusses the practical and analytical considerations for performing interim analysis). | NA |  |  |  | ✔ | ✔ |  | ✔ |  |
| 21 | **Forster et al. 2023 [22]** | Overview article (conference abstract). Discusses the development and implementation of an efficient platform trial design. | NA |  |  | ✔ |  |  |  |  |  |
| 22 | **Gallo 2006 [23]** | Research article (discusses the operational challenges in adaptive design implementation and monitoring). | NA |  |  |  |  | ✔ |  |  |  |
| 23 | **Gallo 2006 [24]** | Research article (discusses confidentiality and trial integrity issues for adaptive designs). | NA |  | ✔ |  |  | ✔ |  |  |  |
| 24 | **Garutti 2014 [25]** | Research article (discusses data challenges in adaptive trials). | NA |  |  |  |  | ✔ |  |  |  |
| 25 | **Gaydos et al. 2009 [26]** | Research article (discusses good practices for adaptive clinical trial in drug development). | NA |  | ✔ | ✔ |  | ✔ |  |  |  |
| 26 | **Gbinigie et al. 2023 [27]** | Study protocol | PANORAMIC, ISRCTN30448031, September 2021 | Platform adaptive trial of novel antivirals for early treatment of COVID-19 in the community. |  | ✔ | ✔ | ✔ | ✔ | ✔ |  |
| 27 | **Geiger et al. 2012 [28]** | Research article | NCT00734474, Phase II/III, August 2008 | An adaptive, dose-finding, seamless study of a long-acting glucagon-like peptide-1 analog (dulaglutide). | ✔ |  |  | ✔ |  |  |  |
| 28 | **Giganti et al. 2025 [29]**  Keshtkar-Jahromi et al. 2023 [30]  ACTIV-6 Study Group 2023 [31]  Lindsell et al. 2024 [32] | Research article (discusses the implementation and challenges encountered in adaptive trials). | ACTIV-2, Phase II/III, (NCT04518410)  August 2020  ACTIV-1, Phase III (NCT04593940)  October 2020  ACTIV-6, Phase III, (NCT04885530), June 2021 | This article discusses the safety and efficacy of multiple investigational agents aimed at modifying the host immune response to SARS-CoV-2 infection or directly enhancing viral control in order to limit disease progression. | ✔ |  | ✔ | ✔ | ✔ | ✔ |  |
| 29 | **Hager et al. 2019 [33]**  Lindsell et al. 2019 [34] | Study protocol | VICTAS, Phase II, NCT03509350, August 2018^a^ | A multi-centre, randomised, placebo-controlled, double-blind, adaptive trial of vitamin C, thiamine and steroids as combination therapy in patients with sepsis. | ✔ | ✔ | ✔ | ✔ |  |  |  |
| 30 | **Hague et al. 2019 [35]**  Brown et al. 2022 [36]  Schiavone et al. 2019 [37] | Research article (discusses implementation and data management challenges encountered in adaptive trials). | FOCUS4 (ISRCTN90061546, July 2013) and STAMPEDE (NCT00268476, July 2005) | Molecularly stratified umbrella trial testing the safety and efficacy of targeted therapies in patients with newly diagnosed metastatic colorectal cancer. FOCUS4 (colorectal cancer) and STAMPEDE (prostate cancer), run from the Medical Research Council Clinical Trials Unit (CTU). | ✔ | ✔ | ✔ | ✔ |  |  | ✔ |
| 31 | **Harkey et al. 2025 [38]** | Research article (discusses process and learnings from HEALY ALS platform trial). | HEALY ALS trial, NCT04297683, NCT04436510, NCT04414345, NCT044364497, Phase II/III, January 2020. | Adaptive platform trial in amyotrophic lateral sclerosis (processes and learnings from the first four regimens of the HEALEY ALS platform trial. |  |  | ✔ | ✔ | ✔ |  |  |
| 32 | **Hayward et al. 2021 [39]** | Study protocol | PRINCIPLE, EudraCT Number 2020-001209-22, Phase III, March 2020 | Platform randomised trial of interventions against COVID-19 in older people. | ✔ |  | ✔ | ✔ |  |  | ✔ |
| 33 | **He et al. 2012 [40]** | Research article (discusses practical considerations and strategies for executing adaptive clinical trials). | NA |  |  | ✔ | ✔ | ✔ |  | ✔ |  |
| 34 | **He et al. 2015 [41]** | Research article (a review of the major developments in the design of adaptive trials in the 21st century). | NA |  |  |  | ✔ |  |  |  |  |
| 35 | **He et al. 2017 [42]** | Research article (addresses challenges and opportunities of ‘‘Less Well-Understood’’ adaptive designs). | NA |  | ✔ |  | ✔ | ✔ |  |  |  |
| 36 | **Holder-Murray et al. 2023 [43]** | Study protocol | REMAP Periop ERP, Phase III, NCT04606264, May 2023 | Randomized, Embedded, Multifactorial Adaptive Platform for Perioperative Medicine at UPMC (UPMC REMAP): Core Protocol - Enhanced Recovery Protocols (ERP) |  |  | ✔ | ✔ | ✔ | ✔ |  |
| 37 | **Huckvale et al. 2023 [44]** | Study protocol | The Vibe Up study, CTRN12621001223820, Phase NS, unknown start date | Protocol for a bandit-based response adaptive trial to evaluate the effectiveness of brief self-guided digital interventions for reducing psychological distress in university students: the Vibe Up study |  |  | ✔ | ✔ |  |  | ✔ |
| 38 | **Hung et al. 2006 [45]** | Research article (discusses regulatory view on adaptive clinical trial design). | NA |  |  |  | ✔ |  |  |  |  |
| 39 | **Huang et al. 2014 [46]** | Research article (discusses the operational and practical issues in planning interim analyses). | NA |  |  |  | ✔ |  |  |  |  |
| 40 | **Kawano-Dourado et al. 2024 [47]** | Research article (discusses implementation of trial). | Trial ID NS, Phase II, trial start date NS | Adaptive multi-interventional trial platform to improve patient care for fibrotic interstitial lung diseases. |  | ✔ | ✔ | ✔ |  | ✔ |  |
| 41 | **Kilbourne et al. 2024 [48]** | Research article (discusses challenges of adaptive trial design implementation). | NA |  |  |  | ✔ |  | ✔ |  |  |
| 42 | **Kim et al. 2025 [49]** | Study protocol | ISRCTN11329596, Phase NS, January 2023 | This trial is part of an adaptive platform trial that forms the testing element of a broader continuous improvement model called ‘IM-SEEN’ (IMprovement Studies for Equitable and Evidence-based iNnovation). |  |  | ✔ | ✔ | ✔ |  |  |
| 43 | **Korn and Freidlin 2017 [50]** | Research/commentary article (discusses advantages and disadvantages of various adaptive design elements). | NA |  |  |  |  | ✔ |  |  |  |
| 44 | **Kotwal et al. 2024 [51]** | Study protocol | NCT06058585 (September 2024) and NCT04389827 (May 2020), Phase III | CAPTIVATE is an international, multi-centre, Phase III, adaptive, platform, randomised controlled trial in people with chronic kidney disease (CKD). |  |  | ✔ |  | ✔ | ✔ |  |
| 45 | **Lauffenburger et al. 2022 [52]** | Research article (discusses important considerations when using adaptive trial designs in health services). | NA |  |  |  |  | ✔ |  |  |  |
| 46 | **Lewis et al. 2011 [53]** | Research article (discusses the design, oversight, and implementation of an adaptive trial). | Phase II, NCT00783081, November 2011^a^ | Randomised, parallel assignment (double masking) to test the safety and efficacy of K-134 for the treatment of intermittent claudication. | ✔ | ✔ | ✔ | ✔ | ✔ |  |  |
| 47 | **Li et al. 2020 [54]** | Research article (discusses the current practice of adaptive design implementation). | NA |  | ✔ |  |  | ✔ |  |  |  |
| 48 | **Lorenzi et al. 2025 [55]** | Research article (discusses the design considerations and statistical considerations in adaptive trial designs). | NA |  |  |  | ✔ |  | ✔ | ✔ |  |
| 49 | **Love et al. 2022. [56]** | Research article (discusses practical recommendations for adaptive designs). | NA |  | ✔ | ✔ | ✔ | ✔ | ✔ |  |  |
| 50 | **Lu et al. 2014 [57]** | Research article (discusses ways of improving efficiency and reducing costs of adaptive trials). | NA |  |  |  | ✔ | ✔ |  |  |  |
| 51 | **Maca et al. 2014 [58]** | Research article (discusses an overview of phase II designs and challenges in adaptive clinical trials). | NA |  |  |  | ✔ |  |  |  |  |
| 52 | **Mahar et al. 2025 [59]** | Commentary | SNAP, NCT05137119), Phase IV, February 2022 | The Staphylococcus aureus Network Adaptive Platform (SNAP) trial is an international multi-centred randomised adaptive platform clinical trial to evaluate a range of interventions to reduce mortality for patients with Staphylococcus Aureus bacteraemia (SAB). |  |  |  |  |  | ✔ |  |
| 53 | **Mahar et al. 2025 [60]** | Research article (discusses the methodological challenges and implementation considerations in adaptive trial designs). | NA |  | ✔ | ✔ | ✔ | ✔ | ✔ | ✔ |  |
| 54 | **Malik et al. 2025 [61]**  Neuhann et al. 2022 [62] | Research articles (discussing methodological approach in VACCELERATE project, including study protocol of a trial conducted within the VACCELERATE network). | VACCELERATE project (including Phase II trial, (EudraCT 2021-004526-29/ NCT05160766), November 2021 | This is a randomised controlled, adaptive, multicentre protocol evaluating different booster strategies in individuals aged 75 years and older already vaccinated against SARS-CoV-2. |  | ✔ | ✔ | ✔ |  | ✔ |  |
| 55 | **Mansberger et al. 2023 [63]** | Research article | The MONT BLANC Study, NCT04445519, Phase III, June 2020 | A adaptive dose selection trial of NCX 470, a nitric oxide-donating bimatoprost for open-angle glaucoma or ocular hypertension. |  |  | ✔ | ✔ | ✔ |  |  |
| 56 | **Mayes et al. 2025 [64]** | Research article | ACCOMPLISH, NCT04829188, Phase NS, March 2021 | Design and methods of an adaptive trial to test comparative effectiveness of  readmission reduction approaches following infection and sepsis  hospitalizations (ACCOMPLISH). |  |  | ✔ | ✔ |  |  |  |
| 57 | **Mehanna et al. 2024 [65]** | Study protocol | ComPARE, CT04116047/ ISRCTN41478539, Phase III, April 2015 | Randomised controlled platform trial comparing alternative regimens for escalating treatment of intermediate and high‑risk oropharyngeal cancer. |  |  | ✔ | ✔ | ✔ |  |  |
| 58 | **Mehta et al. 2010 [66]** | Research article (discusses the methodological, regulatory, and operational issues in implementing interim results). | NA |  | ✔ |  |  |  |  |  |  |
| 59 | **Mehta et al. 2019 [67]** | Research article | TAPPAS trial, NCT02979899, Phase III, February 2017.^a^ | An adaptive population enrichment phase III trial of TRC105 and pazopanib versus pazopanib alone in patients with advanced angiosarcoma (TAPPAS trial). |  |  |  | ✔ |  |  |  |
| 60 | **Metcalfe et al. 2023 [68]** | Study protocol | ISRCTN17825590, May 2018 | Subacromial spacers for adults with symptomatic, irreparable rotator cuff tears: the START:REACTS novel group sequential adaptive randomised controlled trial. |  |  |  |  | ✔ |  |  |
| 61 | **Miller et al. 2014 [69]** | Research article (discusses the different design alternatives in the planning of adaptive trials). | NA |  |  |  | ✔ | ✔ | ✔ |  |  |
| 62 | **Odendaal et al 2023 [70]** | Study protocol | CERM ISRCTN23947730/ EudraCT 2019-000585-38, September 2019 | Preconceptual administration of doxycycline in women with recurrent miscarriage and chronic endometritis: protocol for the Chronic Endometritis and Recurrent Miscarriage (CERM) trial, a multicentre, double-blind, placebo-controlled, adaptive randomised trial with an embedded translational sub study. |  |  |  |  | ✔ |  |  |
| 63 | **Pallmann et al. 2018 [71]** | Research article (provides guidance on key aspects of adaptive designs). | NA |  | ✔ |  | ✔ | ✔ |  |  |  |
| 64 | **Papachristofi et al. 2023 [72]** | Research article (discusses interim decision making in seamless trials designs as applied in an adaptive dose-finding study of a rare kidney disease). | NCT03373461, Phase IIa/b, start date NS | Multicentre, randomised, double-blinded, placebo-controlled, dose-ranging study evaluating safety, tolerability and dose response of a novel compound (iptacopan) in IgA Nephropathy (IgAN) patients. |  |  | ✔ | ✔ |  |  |  |
| 65 | **Park et al. 2020 [73]** | Research article discusses overview of platform trials. | NA |  |  |  | ✔ |  |  |  |  |
| 66 | **Pavlos et al. 2024 [74]** | Study protocol | PATRIC, ACTRN12619000903189, Phase NS, June 2019 | Pragmatic adaptive trial for respiratory infection in children (PATRIC). |  |  |  | ✔ |  |  |  |
| 67 | **Pyle et al. 2024 [75]** | Study protocol | RAPID, ISRCTN33079589, Phase NS, 2022 | Study protocol for an adaptive, multi‑arm, multi‑stage (MAMS) randomised controlled trial of brief remotely delivered psychosocial  interventions for people with serious mental health problems who have experienced a recent suicidal crisis: Remote Approaches to Psychosocial Intervention Delivery (RAPID). |  |  | ✔ | ✔ |  |  |  |
| 68 | **Porcher et al. 2011 [76]** | Research article (discusses when and how adaptive methods should be use in clinical trials). | NA |  |  |  |  | ✔ |  |  |  |
| 69 | **Potvin et al. 2024 [77]** | Research article (discusses current status and future perspective of adaptive designs in dermatology clinical trials). | NA |  |  |  | ✔ | ✔ |  |  |  |
| 70 | **Proschan et al. 2024 [78]** | Research article (discusses  changing interim monitoring in response to internal clinical trial data). | NA |  |  |  |  | ✔ |  |  |  |
| 71 | **Quinlan and Krams 2006 [79]** | Research articles (discusses logistical and operational issues in adaptive clinical trials). | NA |  | ✔ |  | ✔ | ✔ |  |  |  |
| 72 | **Sandercock et al. 2022 [80]** | Research/commentary article (discusses experiences and challenges of the data monitoring committee). | RECOVERY, NCT04381936, Phase II/III, March 2020^a^ | Randomised evaluation of COVID-19 therapy RECOVERY is a randomised trial investigating whether treatment with lopinavir-ritonavir, hydroxychloroquine, corticosteroids, azithromycin, colchicine, IV immunoglobulin (children only), convalescent plasma, casirivimab+Imdevimab, tocilizumab, aspirin, baricitinib, infliximab, empagliflozin, sotrovimab, molnupiravir, paxlovid or anakinra (children only) prevents death in patients with COVID-19.^a^ |  |  |  | ✔ |  |  |  |
| 73 | **Sanchez-Kam et al. 2014 [81]** | Research article (discusses practical guidance in resolving issues related to data monitoring in adaptive trials). | NA |  | ✔ |  |  | ✔ |  |  |  |
| 74 | **Schmdli et al. 2006 [82]** | Research article (discusses practical aspects concerning the planning and implementation of adaptive seamless confirmatory studies). | NA |  |  |  | ✔ |  |  |  |  |
| 75 | **Siskind et al. 2024 [83]** | Study protocol | Cancloz, ACTRN12622001112752, Phase II, 2022 | Protocol for Cancloz: multicentre randomised, placebo-controlled, double-blind, parallel-group adaptive trial of cannabidiol for clozapine-resistant schizophrenia. |  |  | ✔ | ✔ |  |  |  |
| 76 | **Smith et al. 2019 [84]** | Study protocol | Study CGAF, NCT02192190, Phase II, July 2014^a^ | A randomised, double-blind, placebo and active-controlled trial of LY2951742 in participants with mild to moderate osteoarthritis knee pain. |  |  | ✔ | ✔ | ✔ |  |  |
| 77 | **Snowdon et al. 2022 [85]** | Research/commentary article (discusses challenges of operationalising adaptive trials). | UK plasmaMATCH, Phase II, NCT03182634, December 2016^a^ | Umbrella platform trial to assess whether a ctDNA screening can be used to detect patient subgroups who will be sensitive to targeted therapies, and to assess the safety and activity of the targeted treatments.^a^ | ✔ | ✔ |  | ✔ |  |  |  |
| 78 | **Spencer et al. 2012 [86]** | Research article (discusses operational issues with the planning and implementation of adaptive trials and their solutions). | NA |  | ✔ | ✔ | ✔ | ✔ |  | ✔ |  |
| 79 | **Sverdlov et al. 2021 [87]** | Research article (discusses the different types of adaptive designs and review of some regulatory guidelines). | NA |  |  |  | ✔ | ✔ |  |  |  |
| 80 | **Thorlund et al. 2018 [88]** | Research article (discusses design considerations for implementing adaptive trials). | NA |  |  |  | ✔ |  |  |  |  |
| 81 | **Toshner et al. 2022 [89]** | Research article | Phase III, NCT04801940, May 2021^a^ | A national platform trial to evaluate the impact of treatments on longer-term morbidity, mortality, re-hospitalisation, symptom burden and quality of life associated with COVID-19 (HEAL COVID). | ✔ |  | ✔ | ✔ | ✔ |  |  |
| 82 | **VanBuren et al. 2023 [90]** | Research article | Phase IV^a^, NCT01495637 NCT03769844, December 2018^a^ | Nested adaptive clinical trial  of multiple organ dysfunction syndrome  in children. |  |  | ✔ |  |  |  |  |
| 83 | **van Steenwijk et al. 2024 [91]** | Research article | Phase NS, NCT05913622, June 2023 | A randomised embedded multifactorial  adaptive platform for extra corporeal  membrane oxygenation (REMAP ECMO). |  |  |  | ✔ |  |  |  |
| 84 | **Varley et al. 2025 [92]** | Study protocol | Phase II,  ISRCTN53507177, October 2022 | Adaptive clinical trial of AZD7442 and  SARS-CoV-2 vaccination in immunosuppressed patients highly vulnerable to infection with SARS-CoV-2 virus (RAPID-PROTECTION). |  |  |  | ✔ |  |  |  |
| 85 | **Wang et al. 2015. [93]** | Study protocol | Phase IV, NCT01681316, December 2012^a^ | A randomised, double-blind, placebo-controlled trial of danhong injection in the treatment of chronic stable angina.^a^ | ✔ |  | ✔ | ✔ | ✔ |  |  |
| 86 | **Wason et al. 2022 [94]**  Wilson et al. 2021 [95] | Research article (discusses guidance on appropriate resourcing of adaptive trials). | NA |  |  | ✔ |  |  |  |  | ✔ |
| 87 | **Wolstenhulme et al. 2024 [96]** | Study protocol | Phase IIa, ISRCTN81162400, September 2021 | The Graves-PCD trial is a randomised, dose-finding, adaptive trial of the plasma cell-depleting agent daratumumab in severe Graves’ disease. |  |  | ✔ | ✔ |  |  |  |
| 88 | **Yu et al. 2023 [97]** | Research article (discusses implementation and efficient monitoring of adaptive trials). | NA |  | ✔ |  |  |  |  |  |  |
| 89 | **Zahid et al. 2025 [98]** | Study protocol | Quit4TB, Phase II/III, ISRCTN86971818, June 2022 | Adaptive design cluster randomised controlled trial for smoking cessation in people with drug-sensitive pulmonary tuberculosis in Bangladesh and Pakistan. |  |  |  | ✔ |  |  |  |
| 90 | **Zhang et al. 2025 [99]** | Research article | Phase NS, ChiCTR2300071734, start date NS | An adaptive Bayesian randomised controlled trial of traditional Chinese medicine in progressive pulmonary fibrosis. |  |  |  | ✔ |  |  |  |
| 91 | **Zheng et al. 2022 [100]** | Research article (discusses implementation of solutions to the challenges of conducting adaptive trials). | NA |  | ✔ |  | ✔ |  |  | ✔ |  |
| 92 | **Zhu et al. 2023 [101]** | Research article (discusses ADs, their challenges and innovative applications). | NA |  |  |  | ✔ | ✔ |  |  |  |
| Footnotes: a, Information taken from Clinical Trials.gov  Abbreviations: AD, adaptive designs; ctDNA, circulating tumour DNA; NS, not specified; NA, not applicable. | | | | | | | | | | | |

**Reference list of included studies/articles**

1. The Adaptive Platform Trials C. Adaptive platform trials: definition, design, conduct and reporting considerations. Nat Rev Drug Discov. 2019;18(10):797-807. <https://doi.org/10.1038/s41573-019-0034-3>.

2. Aryal D, Beane A, Dondorp A, Green C, Haniffa R, Hashmi M, et al. Operationalisation of the Randomized Embedded Multifactorial Adaptive Platform for COVID-19 trials in a low and lower-middle income critical care learning health system. [version 1; peer review: 3 approved]. Wellcome Open Res. 2021;6:14. <https://doi.org/10.12688/wellcomeopenres.16486.1>.

3. Huang DT, McVerry BJ, Horvat C, Adams PW, Berry S, Buxton M, et al. Implementation of the Randomized Embedded Multifactorial Adaptive Platform for COVID-19 (REMAP-COVID) trial in a US health system—lessons learned and recommendations. Trials. 2021;22(1):100. <https://doi.org/10.1186/s13063-020-04997-6>.

4. Bhatt DL, Mehta CR. Adaptive Designs for Clinical Trials. New England journal of medicine. 2016;375(1):65-74. <https://doi.org/10.1056/nejmra1510061>.

5. Bauer P, Bretz F, Dragalin V, König F, Wassmer G. Twenty-five years of confirmatory adaptive designs: opportunities and pitfalls. Stat Med. 2016;35(3):325-47. <https://doi.org/10.1002/sim.6472>.

6. Bender BG, Cvietusa PJ, Goodrich GK, King DK, Shoup JA. Adapting adaptive design methods to accelerate adoption of a digital asthma management intervention. Transl Behav Med. 2023;13(3):149-55. <https://doi.org/10.1093/tbm/ibac093>.

7. Bretz F, Branson M, Burman C-F, Chuang-Stein C, Coffey CS. Adaptivity in drug discovery and development. Drug Dev Res. 2009;70(3):169-90. <https://doi.org/10.1002/ddr.20285>.

8. Brown AR, Gajewski BJ, Mudaranthakam DP, Pasnoor M, Dimachkie MM, Jawdat O, et al. Conducting a bayesian multi-armed trial with response adaptive randomization for comparative effectiveness of medications for CSPN. Contemp Clin Trials Commun. 2023;36:101220. <https://doi.org/https://doi.org/10.1016/j.conctc.2023.101220>.

9. Bwakura-Dangarembizi M, Amadi B, Singa BO, Muyemayema S, Ngosa D, Mwalekwa L, et al. An adaptive multiarm randomised trial of biomedical and psychosocial interventions to improve convalescence following severe acute malnutrition in sub-Saharan Africa: Co-SAM trial protocol. BMJ Open. 2025;15(5):e093758. <https://doi.org/10.1136/bmjopen-2024-093758>.

10. Chen DZ, Patel SS, Xie A, Chen J, Castle D, Ma C. RaBIt: An Effective Visualization-Driven Tool for Power and Sample Size Estimation in Two-Stage General Randomized Basket Trial Designs. medRxiv. 2025:2024.09.19.24313989. <https://doi.org/10.1101/2024.09.19.24313989>.

11. Chen YHJ, Gesser R, Luxembourg A. A seamless Phase IIB/III adaptive outcome trial: Design rationale and implementation challenges. Clin Trials. 2015;12(1):84-90. <https://doi.org/10.1177/1740774514552110>.

12. Chow S-C, Corey R, Lin M. On the Independence of Data Monitoring Committee in Adaptive Design Clinical Trials. J Biopharm Stat. 2012;22(4):853-67. <https://doi.org/10.1080/10543406.2012.676536>.

13. Cook TD, Benner RJ, Fisher MR. The WIZARD Trial as a Case Study of Flexible Clinical Trial Design. Drug Inf J. 2006;40(3):345-53. <https://doi.org/10.1177/009286150604000313>.

14. Currie GE, Totterdell J, Bowland G, Leeb A, Peters I, Blyth CC, et al. The AuTOMATIC trial: a study protocol for a multi-arm Bayesian adaptive randomised controlled trial of text messaging to improve childhood immunisation coverage. Trials. 2023;24(1):97. <https://doi.org/10.1186/s13063-023-07097-3>.

15. Dawson SN, Chiu Y-D, Klein AA, Earwaker M, Villar SS, Duckworth M, et al. Effect of high-flow nasal therapy on patient-centred outcomes in patients at high risk of postoperative pulmonary complications after cardiac surgery: update to the statistical analysis plan for NOTACS, a multicentre adaptive randomised controlled trial. Trials. 2024;25(1):741. <https://doi.org/10.1186/s13063-024-08538-3>.

16. DeMets DL, Wittes J. Data monitoring committee interim reports: We must get there soon! Clin Trials. 2022;19(1):107-11. <https://doi.org/10.1177/17407745211051279>.

17. Dey M, Pyle L. Managing Operational Challenges for Interim Analyses. In: Pharmaceutical SAS Users Group conference proceedings, June 1-4, Atlanta, Georgia. 2008. <https://www.lexjansen.com/pharmasug/2008/po/PO21.pdf>.

18. Fabbri M, Rascol O, Foltynie T, Carroll C, Postuma RB, Porcher R, et al. Advantages and Challenges of Platform Trials for Disease Modifying Therapies in Parkinson's Disease. Mov Disord. 2024;39(9):1468-77. <https://doi.org/https://doi.org/10.1002/mds.29899>.

19. Fardipour P, Littman G, Burns DD, Dragalin V, Padmanabhan SK, Parke T, et al. Planning and Executing Response-Adaptive Learn-Phase Clinical Trials: 1. The Process. Drug Inf J. 2009;43(6):713-23. <https://doi.org/10.1177/009286150904300609>.

20. Files DC, Matthay MA, Calfee CS, Aggarwal NR, Asare AL, Beitler JR, et al. I-SPY COVID adaptive platform trial for COVID-19 acute respiratory failure: rationale, design and operations. BMJ Open. 2022;12(6):e060664. <https://doi.org/10.1136/bmjopen-2021-060664>.

21. Fleming S, Mwandigha L, Fanshawe TR. Practical and analytical considerations when performing interim analyses in diagnostic test accuracy studies. Diagnostic and Prognostic Research. 2024;8(1):12. <https://doi.org/10.1186/s41512-024-00174-4>.

22. Forster A, Moreau L, Wright-Hughes A, Schofield H, Day F, Murden G, et al. Developing a platform for delivering effecient trials for people living with stroke: The life after stroke platform (LEAP). Poster presentation at the 15th World Stroke Congress, 10-12 October 2023, Toronto, Canada. Int J Stroke. 2023;18(3_suppl):3-420. <https://doi.org/10.1177/17474930231192010>.

23. Gallo P. Operational challenges in adaptive design implementation. Pharm Stat. 2006;5(2):119-24. <https://doi.org/10.1002/pst.221>.

24. Gallo P. Confidentiality and Trial Integrity Issues for Adaptive Designs. Drug Inf J. 2006;40(4):445-50. <https://doi.org/10.1177/216847900604000410>.

25. Garutti C. Data Challenges in Adaptive Trials. 2014. Available from: <https://www.lexjansen.com/phuse/2014/dh/DH04.pdf>. Accessed 14 June 2024.

26. Gaydos B, Anderson KM, Berry D, Burnham N, Chuang-Stein C, Dudinak J, et al. Good Practices for Adaptive Clinical Trials in Pharmaceutical Product Development. Drug Inf J. 2009;43(5):539-56. <https://doi.org/10.1177/009286150904300503>.

27. Gbinigie O, Ogburn E, Allen J, Dorward J, Dobson M, Madden T-A, et al. Platform adaptive trial of novel antivirals for early treatment of COVID-19 In the community (PANORAMIC): protocol for a randomised, controlled, open-label, adaptive platform trial of community novel antiviral treatment of COVID-19 in people at increased risk of more severe disease. BMJ Open. 2023;13(8):e069176. <https://doi.org/10.1136/bmjopen-2022-069176>.

28. Geiger MJ, Skrivanek Z, Gaydos B, Chien J, Berry S, Berry D, et al. An Adaptive, Dose-Finding, Seamless Phase 2/3 Study of a Long-Acting Glucagon-like Peptide-1 Analog (Dulaglutide): Trial Design and Baseline Characteristics. J Diabetes Sci Technol. 2012;6(6):1319-27. <https://doi.org/10.1177/193229681200600610>.

29. Giganti MJ, Chew KW, Moser C, Eron JJ, Pinilla M, Li JZ, et al. Implementation of a seamless phase 2/3 study design in the setting of an emergent infectious disease pandemic: Lessons learned from the ACTIV-2 platform COVID-19 treatment trial. Contemp Clin Trials. 2025;153:107887. <https://doi.org/https://doi.org/10.1016/j.cct.2025.107887>.

30. Keshtkar-Jahromi M, Adam SJ, Brar I, Chung LK, Currier JS, Daar ES, et al. ACTIV trials: cross-trial lessons learned for master protocol implementation. Journal of Clinical and Translational Science. 2024;8(1):e152. <https://doi.org/10.1017/cts.2024.507>.

31. The Accelerating Covid-19 Therapeutic Interventions and Vaccines (ACTIV)-6 Study Group. ACTIV-6: Operationalizing a decentralized, outpatient randomized platform trial to evaluate efficacy of repurposed medicines for COVID-19. Journal of Clinical and Translational Science. 2023;7(1):e221. <https://doi.org/10.1017/cts.2023.644>.

32. Lindsell CJ, Shotwell M, Anstrom KJ, Berry S, Brittain E, Harrell FE, et al. The statistical design and analysis of pandemic platform trials: Implications for the future. Journal of Clinical and Translational Science. 2024;8(1):e155. <https://doi.org/10.1017/cts.2024.514>.

33. Hager DN, Hooper MH, Bernard GR, Busse LW, Ely EW, Fowler AA, et al. The Vitamin C, Thiamine and Steroids in Sepsis (VICTAS) Protocol: a prospective, multi-center, double-blind, adaptive sample size, randomized, placebo-controlled, clinical trial. Trials. 2019;20(1):197. <https://doi.org/10.1186/s13063-019-3254-2>.

34. Lindsell CJ, McGlothlin A, Nwosu S, Rice TW, Hall A, Bernard GR, et al. Update to the Vitamin C, Thiamine and Steroids in Sepsis (VICTAS) protocol: statistical analysis plan for a prospective, multicenter, double-blind, adaptive sample size, randomized, placebo-controlled, clinical trial. Trials. 2019;20(1):670. <https://doi.org/10.1186/s13063-019-3775-8>.

35. Hague D, Townsend S, Masters L, Rauchenberger M, Van Looy N, Diaz-Montana C, et al. Changing platforms without stopping the train: experiences of data management and data management systems when adapting platform protocols by adding and closing comparisons. Trials. 2019;20(1):294. <https://doi.org/10.1186/s13063-019-3322-7>.

36. Brown LC, Graham J, Fisher D, Adams R, Seligmann J, Seymour M, et al. Experiences of running a stratified medicine adaptive platform trial: Challenges and lessons learned from 10 years of the FOCUS4 trial in metastatic colorectal cancer. Clin Trials. 2022;19(2):146-57. <https://doi.org/10.1177/17407745211069879>.

37. Schiavone F, Bathia R, Letchemanan K, Masters L, Amos C, Bara A, et al. This is a platform alteration: a trial management perspective on the operational aspects of adaptive and platform and umbrella protocols. Trials. 2019;20(1):264. <https://doi.org/10.1186/s13063-019-3216-8>.

38. Harkey BA, Distefano S, Pagliaro JA, Heyd L, Chase M, Igne C, et al. Operational Development and Launch of an Adaptive Platform Trial in Amyotrophic Lateral Sclerosis: Processes and Learnings From the First Four Regimens of the HEALEY ALS Platform Trial. Muscle Nerve. 2025;72(2):294-305. <https://doi.org/https://doi.org/10.1002/mus.28442>.

39. Hayward G, Butler CC, Yu L-M, Saville BR, Berry N, Dorward J, et al. Platform Randomised trial of INterventions against COVID-19 In older peoPLE (PRINCIPLE): protocol for a randomised, controlled, open-label, adaptive platform, trial of community treatment of COVID-19 syndromic illness in people at higher risk. BMJ Open. 2021;11(6):e046799. <https://doi.org/10.1136/bmjopen-2020-046799>.

40. He W, Kuznetsova OM, Harmer M, Leahy C, Anderson K, Dossin N, et al. Practical Considerations and Strategies for Executing Adaptive Clinical Trials. Drug Inf J. 2012;46(2):160-74. <https://doi.org/10.1177/0092861512436580>.

41. He P, Lai TL, Su Z. Design of clinical trials with failure-time endpoints and interim analyses: An update after fifteen years. Contemp Clin Trials. 2015;45(Part A):103-12. <https://doi.org/10.1016/j.cct.2015.05.018>.

42. He W, Gallo P, Miller E, Jemiai Y, Maca J, Koury K, et al. Addressing Challenges and Opportunities of "Less Well-Understood" Adaptive Designs. Ther Innov Regul Sci. 2017;51(1):60-8. <https://doi.org/10.1177/2168479016663265>.

43. Holder-Murray J, Esper SA, Althans AR, Knight J, Subramaniam K, Derenzo J, et al. REMAP Periop: a randomised, embedded, multifactorial adaptive platform trial protocol for perioperative medicine to determine the optimal enhanced recovery pathway components in complex abdominal surgery patients within a US healthcare system. BMJ Open. 2023;13(12):e078711. <https://doi.org/10.1136/bmjopen-2023-078711>.

44. Huckvale K, Hoon L, Stech E, Newby JM, Zheng WY, Han J, et al. Protocol for a bandit-based response adaptive trial to evaluate the effectiveness of brief self-guided digital interventions for reducing psychological distress in university students: the Vibe Up study. BMJ Open. 2023;13(4):e066249. <https://doi.org/10.1136/bmjopen-2022-066249>.

45. Hung HMJ, O'Neill RT, Wang S-J, Lawrence J. A Regulatory View on Adaptive/Flexible Clinical Trial Design. Biom J. 2006;48(4):565-73. <https://doi.org/10.1002/bimj.200610229>.

46. Huang J, Das A, Burger HU, Zhong W, Zhang W, Lieberman G. The use of phase 2 interim analysis to expedite drug development decisions. Contemp Clin Trials. 2014;38(2):235-44. <https://doi.org/10.1016/j.cct.2014.05.006>.

47. Kawano-Dourado L, Kulkarni T, Ryerson CJ, Rivera-Ortega P, Baldi BG, Chaudhuri N, et al. Adaptive multi-interventional trial platform to improve patient care for fibrotic interstitial lung diseases. Thorax. 2024;79(8):788. <https://doi.org/10.1136/thorax-2023-221148>.

48. Kilbourne A, Chinman M, Rogal S, Almirall D. Adaptive Designs in Implementation Science and Practice: Their Promise and the Need for Greater Understanding and Improved Communication. Annu Rev Public Health. 2024;45(Volume 45, 2024):69-88. <https://doi.org/https://doi.org/10.1146/annurev-publhealth-060222-014438>.

49. Kim M, Allen L, Tlhajoane M, Prieto-Merino D, Bolster N, Bastawrous A, et al. Enhanced patient counselling and SMS reminder messages to improve access to community-based eye care services in Meru, Kenya: statistical analysis plan for a Bayesian adaptive trial [version 1; peer review: 2 approved]. Wellcome Open Res. 2025;10(69). <https://doi.org/10.12688/wellcomeopenres.23495.1>.

50. Korn EL, Freidlin B. Adaptive Clinical Trials: Advantages and Disadvantages of Various Adaptive Design Elements. J Natl Cancer Inst. 2017;109(6):djx013. <https://doi.org/10.1093/jnci/djx013>.

51. Kotwal SS, Perkovic V, Jardine MJ, Kim D, Shah NA, Lin E, et al. The Global Kidney Patient Trials Network and the CAPTIVATE Platform Clinical Trial Design: A Trial Protocol. JAMA Network Open. 2024;7(12):e2449998-e. <https://doi.org/10.1001/jamanetworkopen.2024.49998>.

52. Lauffenburger JC, Choudhry NK, Russo M, Glynn RJ, Ventz S, Trippa L. Designing and conducting adaptive trials to evaluate interventions in health services and implementation research: practical considerations. BMJ Med. 2022;1(1):e000158. <https://doi.org/10.1136/bmjmed-2022-000158>.

53. Lewis RJ, Connor JT, Teerlink JR, Murphy JR, Cooper LT, Hiatt WR, et al. Application of adaptive design and decision making to a phase II trial of a phosphodiesterase inhibitor for the treatment of intermittent claudication. Trials. 2011;12(1):134. <https://doi.org/10.1186/1745-6215-12-134>.

54. Li Q, Lin J, Lin Y. Adaptive design implementation in confirmatory trials: methods, practical considerations and case studies. Contemp Clin Trials. 2020;98:106096. <https://doi.org/10.1016/j.cct.2020.106096>.

55. Lorenzi E, Crawford AM, Anderson CS, Menon B, Chen X, Mistry E, et al. Adaptive Platform Trials in Stroke. Stroke. 2025;56(1):198-208. <https://doi.org/10.1161/STROKEAHA.124.045754>.

56. Love SB, Cafferty F, Snowdon C, Carty K, Savage J, Pallmann P, et al. Practical guidance for running late-phase platform protocols for clinical trials: lessons from experienced UK clinical trials units. Trials. 2022;23(1):757. <https://doi.org/10.1186/s13063-022-06680-4>.

57. Lu M, Ownby DR, Zoratti E, Roblin D, Johnson D, Johnson CC, et al. Improving efficiency and reducing costs: Design of an adaptive, seamless, and enriched pragmatic efficacy trial of an online asthma management program. Contemp Clin Trials. 2014;38(1):19-27. <https://doi.org/10.1016/j.cct.2014.02.008>.

58. Maca J, Dragalin V, Gallo P. Adaptive Clinical Trials: Overview of Phase III Designs and Challenges. Ther Innov Regul Sci. 2014;48(1):31-40. <https://doi.org/10.1177/2168479013507436>.

59. Mahar RK, McGlothlin A, Dymock M, Barina L, Bonten M, Bowen A, et al. Statistical documentation for multi-disease, multi-domain platform trials: our experience with the Staphylococcus aureus Network Adaptive Platform trial. Trials. 2025;26(1):49. <https://doi.org/10.1186/s13063-024-08684-8>.

60. Mahar R, Webb S, Marschner I, Forbes AB, Lee KJ. Platform trials: key features, when to use them and methodological challenges. Med J Aust. 2025;223(3):120-2. <https://doi.org/https://doi.org/10.5694/mja2.52711>.

61. Malik S, Dorothea ZP, Argyropoulos CD, Themistocleous S, Macken AJ, Valdenmaiier O, et al. Data Interoperability in COVID-19 Vaccine Trials: Methodological Approach in the VACCELERATE Project. JMIR Med Inform. 2025;13:e65590. <https://doi.org/10.2196/65590>.

62. Neuhann JM, Stemler J, Carcas A, Frías-Iniesta J, Bethe U, Heringer S, et al. A multinational, phase 2, randomised, adaptive protocol to evaluate immunogenicity and reactogenicity of different COVID-19 vaccines in adults ≥75 already vaccinated against SARS-CoV-2 (EU-COVAT-1-AGED): a trial conducted within the VACCELERATE network. Trials. 2022;23(1):865. <https://doi.org/10.1186/s13063-022-06791-y>.

63. Mansberger SL, Fechtner R, Lopez K, Hubatsch D. A phase 3 adaptive dose selection trial of NCX 470, a nitric oxide-donating bimatoprost for open-angle glaucoma or ocular hypertension: The MONT BLANC study. Contemp Clin Trials. 2024;147:107730. <https://doi.org/https://doi.org/10.1016/j.cct.2024.107730>.

64. Mayes K, Talisa VB, Malito A, Mayr FB, Williams K, Char K, et al. Design and methods of an adaptive trial to test comparative effectiveness of readmission reduction approaches following infection and sepsis hospitalizations (ACCOMPLISH). Contemp Clin Trials Commun. 2025;46:101504. <https://doi.org/https://doi.org/10.1016/j.conctc.2025.101504>.

65. Mehanna H, Gaunt P, Kong A, Hartley A, Sanghera P, Forster M, et al. CompARE: study protocol for a phase III randomised controlled platform trial comparing alternative regimens for escalating treatment of intermediate and high-risk oropharyngeal cancer. Trials. 2024;25(1):50. <https://doi.org/10.1186/s13063-023-07881-1>.

66. Mehta CR, Pocock SJ. Adaptive increase in sample size when interim results are promising: a practical guide with examples. Stat Med. 2010;30(28):3267-84. <https://doi.org/10.1002/sim.4102>.

67. Mehta CR, Liu L, Theuer C. An adaptive population enrichment phase III trial of TRC105 and pazopanib versus pazopanib alone in patients with advanced angiosarcoma (TAPPAS trial). Ann Oncol. 2019;30(1):103-8. <https://doi.org/10.1093/annonc/mdy464>.

68. Metcalfe A, Arnold S, Parsons H, Parsons N, Bhabra G, Brown J, et al. Subacromial spacers for adults with symptomatic, irreparable rotator cuff tears: the START:REACTS novel group sequential adaptive RCT. 2023;10:03. <https://doi.org/10.3310/TKJY2101>.

69. Miller F, Björnsson M, Svensson O, Karlsten R. Experiences with an adaptive design for a dose-finding study in patients with osteoarthritis. Contemp Clin Trials. 2014;37(2):189-99. <https://doi.org/10.1016/j.cct.2013.12.007>.

70. Odendaal J, Black N, Bouliotis G, Guck J, Underwood M, Fisher J, et al. Preconceptual administration of doxycycline in women with recurrent miscarriage and chronic endometritis: protocol for the Chronic Endometritis and Recurrent Miscarriage (CERM) trial, a multicentre, double-blind, placebo-controlled, adaptive randomised trial with an embedded translational substudy. BMJ Open. 2023;13(12):e081470. <https://doi.org/10.1136/bmjopen-2023-081470>.

71. Pallmann P, Bedding A, Choodari-Oskooei B, Dimairo M, Flight L, Hampson LV, et al. Adaptive designs in clinical trials: why use them, and how to run and report them. BMC Med. 2018;16:29. <https://doi.org/10.1186/s12916-018-1017-7>.

72. Papachristofi O, Bornkamp B, Wright M, Friede T. Interim decision making in seamless trial designs: An application in an adaptive dose-finding study in a rare kidney disease. Pharm Stat. 2023;23(1):20-30. <https://doi.org/10.1002/pst.2335>.

73. Park JJH, Harari O, Dron L, Lester RT, Thorlund K, Mills EJ. An overview of platform trials with a checklist for clinical readers. J Clin Epidemiol. 2020;125:1-8. <https://doi.org/10.1016/j.jclinepi.2020.04.025>.

74. Pavlos R, Bhuiyan MU, Jones M, Oakes D, Brien S, Borland ML, et al. Pragmatic Adaptive Trial for Respiratory Infection in Children (PATRIC) Clinical Registry protocol. BMJ Open. 2024;14(1):e074308. <https://doi.org/10.1136/bmjopen-2023-074308>.

75. Pyle M, Loftus L, Emsley R, Freeman D, Gillard S, Gumley A, et al. Study protocol for an adaptive, multi-arm, multi-stage (MAMS) randomised controlled trial of brief remotely delivered psychosocial interventions for people with serious mental health problems who have experienced a recent suicidal crisis: Remote Approaches to Psychosocial Intervention Delivery (RAPID). Trials. 2024;25(1):460. <https://doi.org/10.1186/s13063-024-08293-5>.

76. Porcher R, Lecocq B, Vray M, d’Andon A, Bassompierre F, Béhier J-M, et al. Adaptive Methods: When and How Should They be Used in Clinical Trials? Therapies. 2011;66(4):319-26. <https://doi.org/10.2515/therapie/2011044>.

77. Potvin D, D'Angelo P, Bennett S, Jankicevic J, Bissonnette R. Adaptive designs in dermatology clinical trials: Current status and future perspectives. J Eur Acad Dermatol Venereol. 2024;38(9):1694-703. <https://doi.org/https://doi.org/10.1111/jdv.20030>.

78. Proschan MA, Nason M, Ortega-Villa AM, Wang J. Changing interim monitoring in response to internal clinical trial data. Biometrics. 2024;80(1):ujae006. <https://doi.org/10.1093/biomtc/ujae006>.

79. Quinlan J, Krams M. Implementing Adaptive Designs: Logistical and Operational Considerations. Drug Inf J. 2006;40(4):437-44. <https://doi.org/10.1177/216847900604000409>.

80. Sandercock PAG, Darbyshire J, DeMets D, Fowler R, Lalloo DG, Munavvar M, et al. Experiences of the Data Monitoring Committee for the RECOVERY trial, a large-scale adaptive platform randomised trial of treatments for patients hospitalised with COVID-19. Trials. 2022;23(1):881. <https://doi.org/10.1186/s13063-022-06824-6>.

81. Sanchez-Kam M, Gallo P, Loewy J, Menon S, Antonijevic Z, Christensen J, et al. A Practical Guide to Data Monitoring Committees in Adaptive Trials. Ther Innov Regul Sci. 2014;48(3):316-26. <https://doi.org/10.1177/2168479013509805>.

82. Schmidli H, Bretz F, Racine A, Maurer W. Confirmatory Seamless Phase II/III Clinical Trials with Hypotheses Selection at Interim: Applications and Practical Considerations. Biom J. 2006;48(4):635-43. <https://doi.org/10.1002/bimj.200510231>.

83. Siskind D, Bull C, Suetani S, Warren N, Suraev A, McGregor I, et al. Protocol for Cancloz: multicentre randomised, placebo-controlled, double-blind, parallel-group adaptive trial of cannabidiol for clozapine-resistant schizophrenia. BJPsych Open. 2024;10(5):e156. <https://doi.org/10.1192/bjo.2024.748>.

84. Smith CL, Jin Y, Raddad E, McNearney TA, Ni X, Monteith D, et al. Applications of Bayesian statistical methodology to clinical trial design: A case study of a phase 2 trial with an interim futility assessment in patients with knee osteoarthritis. Pharm Stat. 2019;18(1):39-53. <https://doi.org/10.1002/pst.1906>.

85. Snowdon C, Kernaghan S, Moretti L, Turner NC, Ring A, Wilkinson K, et al. Operational complexity versus design efficiency: challenges of implementing a phase IIa multiple parallel cohort targeted treatment platform trial in advanced breast cancer. Trials. 2022;23(1):372. <https://doi.org/10.1186/s13063-022-06312-x>.

86. Spencer K, Colvin K, Braunecker B, Brackman M, Ripley J, Hines P, et al. Operational Challenges and Solutions with Implementation of an Adaptive Seamless Phase 2/3 Study. J Diabetes Sci Technol. 2012;6(6):1296-304. <https://doi.org/10.1177/193229681200600608>.

87. Sverdlov O, Ryeznik Y, Wong WK. Opportunity for efficiency in clinical development: An overview of adaptive clinical trial designs and innovative machine learning tools, with examples from the cardiovascular field. Contemp Clin Trials. 2021;105:106397. <https://doi.org/10.1016/j.cct.2021.106397>.

88. Thorlund K, Haggstrom J, Park JJ, Mills EJ. Key design considerations for adaptive clinical trials: a primer for clinicians. BMJ. 2018;360:k698. <https://doi.org/10.1136/bmj.k698>.

89. Toshner MR, Gamble C, Baillie JK, Best A, Bedson E, Bradley J, et al. Apixaban following discharge in hospitalised adults with COVID-19: Preliminary results from a multicentre, open-label, randomised controlled platform clinical trial. medRxiv. 2022. <https://doi.org/10.1101/2022.12.07.22283175>.

90. VanBuren JM, Hall M, Zuppa AF, Mourani PM, Carcillo J, Dean JM, et al. The Design of Nested Adaptive Clinical Trials of Multiple Organ Dysfunction Syndrome Children in a Single Study. Pediatr Crit Care Med. 2023;24(12).

91. van Steenwijk MPJ, van Rosmalen J, Elzo Kraemer CV, Donker DW, Hermens JAJM, Kraaijeveld AO, et al. A randomized embedded multifactorial adaptive platform for extra corporeal membrane oxygenation (REMAP ECMO) – design and rationale of the left ventricular unloading trial domain. Am Heart J. 2025;279:81-93. <https://doi.org/https://doi.org/10.1016/j.ahj.2024.10.010>.

92. Varley M, Euden J, Adams R, Barnes E, Bodman C, Choy E, et al. Adaptive clinical trial of AZD7442 and SARS-CoV-2 vaccination in immunosuppressed patients highly vulnerable to infection with SARS-CoV-2 virus (RAPID-PROTECTION): protocol for a multicentre, interventional open-label, randomised controlled trial. BMJ Open. 2025;15(1):e084345. <https://doi.org/10.1136/bmjopen-2024-084345>.

93. Wang PQ, Li DD, Dong W, Liu J, Yu YN, Shen CT, et al. Danhong injection in the treatment of chronic stable angina: study protocol for a randomized controlled trial. Trials. 2015;16(1):474. <https://doi.org/10.1186/s13063-015-0998-1>.

94. Wason JMS, Dimairo M, Biggs K, Bowden S, Brown J, Flight L, et al. Practical guidance for planning resources required to support publicly-funded adaptive clinical trials. BMC Med. 2022;20:254. <https://doi.org/10.1186/s12916-022-02445-7>.

95. Wilson N, Biggs K, Bowden S, Brown J, Dimairo M, Flight L, et al. Costs and staffing resource requirements for adaptive clinical trials: quantitative and qualitative results from the Costing Adaptive Trials project. BMC Med. 2021;19:251. <https://doi.org/10.1186/s12916-021-02124-z>.

96. Wolstenhulme F, Bibby I, Cole M, Grixti L, McGregor N, Bradley P, et al. Graves-PCD: protocol for a randomised, dose-finding, adaptive trial of the plasma cell-depleting agent daratumumab in severe Graves’ disease. BMJ Open. 2024;14(6):e079158. <https://doi.org/10.1136/bmjopen-2023-079158>.

97. Yu Z, Wu L, Bunn V, Li Q, Lin J. Evolution of Phase II Oncology Trial Design: from Single Arm to Master Protocol. Ther Innov Regul Sci. 2023;57(4):823-38. <https://doi.org/10.1007/s43441-023-00500-w>.

98. Zahid M, Rahman F, Chowdhury A, Rana SH, Ansaari S, Lim AK, et al. mHealth intervention (mTB-Tobacco) for smoking cessation in people with drug-sensitive pulmonary tuberculosis in Bangladesh and Pakistan: protocol for an adaptive design, cluster randomised controlled trial (Quit4TB). BMJ Open. 2025;15(2):e089007. <https://doi.org/10.1136/bmjopen-2024-089007>.

99. Zhang C, Nie Y-s, Zhang C-t, Yang H-j, Zhang H-r, Xiao W, et al. An adaptive Bayesian randomized controlled trial of traditional Chinese medicine in progressive pulmonary fibrosis: Rationale and study design. Journal of Integrative Medicine. 2025;23(2):138-44. <https://doi.org/https://doi.org/10.1016/j.joim.2025.01.003>.

100. Zheng R, Ito YM, Yunoki M, Minoda K, Nobeyama S. Design and implementation of an adaptive confirmatory trial in Japanese patients with palmoplantar pustulosis. Contemp Clin Trials Commun. 2022;28:100935. <https://doi.org/10.1016/j.conctc.2022.100935>.

101. Zhu H, Wong WK. An Overview of Adaptive Designs and Some of Their Challenges, Benefits, and Innovative Applications. J Med Internet Res. 2023;25:e44171. <https://doi.org/10.2196/44171>.
